# Functional and evolutionary analyses of *Helicobacter pylori* HP0231 (DsbK) protein with strong oxidative and chaperone activity characterized by a highly diverged dimerization domain

**DOI:** 10.3389/fmicb.2015.01065

**Published:** 2015-10-08

**Authors:** Katarzyna M. Bocian-Ostrzycka, Anna M. Łasica, Stanisław Dunin-Horkawicz, Magdalena J. Grzeszczuk, Karolina Drabik, Aneta M. Dobosz, Renata Godlewska, Elżbieta Nowak, Jean-Francois Collet, Elżbieta K. Jagusztyn-Krynicka

**Affiliations:** ^1^Department of Bacterial Genetics, Institute of Microbiology, Faculty of Biology, University of WarsawWarsaw, Poland; ^2^Laboratory of Bioinformatics and Protein Engineering, International Institute of Molecular and Cell BiologyWarsaw, Poland; ^3^Laboratory of Protein Structure, International Institute of Molecular and Cell BiologyWarsaw, Poland; ^4^de Duve Institute, Université catholique de Louvain (UCL)/Walloon Excellence in Life Sciences and BiotechnologyBrussels, Belgium

**Keywords:** *Helicobacter pylori*, disulfide bonds, Dsb proteins, oxidoreductase activity, chaperone activity

## Abstract

*Helicobacter pylori* does not encode the classical DsbA/DsbB oxidoreductases that are crucial for oxidative folding of extracytoplasmic proteins. Instead, this microorganism encodes an untypical two proteins playing a role in disulfide bond formation – periplasmic HP0231, which structure resembles that of EcDsbC/DsbG, and its redox partner, a membrane protein HpDsbI (HP0595) with a β-propeller structure. The aim of presented work was to assess relations between HP0231 structure and function. We showed that HP0231 is most closely related evolutionarily to the catalytic domain of DsbG, even though it possesses a catalytic motif typical for canonical DsbA proteins. Similarly, the highly diverged N-terminal dimerization domain is homologous to the dimerization domain of DsbG. To better understand the functioning of this atypical oxidoreductase, we examined its activity using *in vivo* and *in vitro* experiments. We found that HP0231 exhibits oxidizing and chaperone activities but no isomerizing activity, even though *H. pylori* does not contain a classical DsbC. We also show that HP0231 is not involved in the introduction of disulfide bonds into HcpC (*Helicobacter* cysteine-rich protein C), a protein involved in the modulation of the *H. pylori* interaction with its host. Additionally, we also constructed a truncated version of HP0231 lacking the dimerization domain, denoted HP0231m, and showed that it acts in *Escherichia coli* cells in a DsbB-dependent manner. In contrast, HP0231m and classical monomeric EcDsbA (*E. coli* DsbA protein) were both unable to complement the lack of HP0231 in *H. pylori* cells, though they exist in oxidized forms. HP0231m is inactive in the insulin reduction assay and possesses high chaperone activity, in contrast to EcDsbA. In conclusion, HP0231 combines oxidative functions characteristic of DsbA proteins and chaperone activity characteristic of DsbC/DsbG, and it lacks isomerization activity.

## Introduction

In many Gram-negative bacteria, the periplasmic space is the major site for the maturation of proteins that enter this compartment. The three-dimensional structure of proteins is crucial for their function and stability. The proper folding of secreted proteins containing cysteine residues, especially those with an even number of cysteines, often requires the oxidation of thiol groups between two cysteine residues and the formation of a covalent bond. Several excellent reviews about functioning of the *Escherichia coli* Dsb system have recently been published (Kadokura and Beckwith, 2010; Depuydt et al., 2011; Berkmen, 2012; Denoncin and Collet, 2013; Hatahet et al., 2014).

In *E. coli* and other Gram-negative bacteria, the extracytoplasmic generation of disulfide bonds is catalyzed by disulfide oxidoreductases referred to as DsbAs. These proteins are usually periplasmic, monomeric, soluble proteins that structurally belong to the thioredoxin-superfamily. EcDsbA (a 21-kDa monomeric protein) directly donates its disulfide bond present in the active site to unfolded, reduced protein substrates (Paxman et al., 2009). A strictly conserved CXXC catalytic motif (CPHC in EcDsbA) present within the TRX (*thioredoxin*) fold is required for activity as well as a proline residue, always found in *cis*-conformation. The loop region containing the *cis*-proline residue, although distant from the CXXC active site in the linear sequence but close in the three-dimensional structure, is important for maintaining both the conformation of the active site and the redox potential of the protein (Kadokura et al., 2005; Ren et al., 2009). EcDsbA activity is dependent on the action of the inner membrane protein DsbB, which restores it to the original, oxidized form. Classical EcDsbB, protein with four transmembrane segments, encompasses two pairs of conserved, catalytic cysteine residues located in the periplasmic loops. The process depends on the cell respiratory chain (Inaba, 2008).

The action of DsbA is an error-prone process because DsbA preferentially introduces disulfide bonds between cysteines that are consecutive in the primary sequence of the protein (Inaba, 2010). Thus, the proteins whose proper folding requires the formation of disulfide bonds between cysteines that are non-consecutive in the sequence often end up misoxidized and misfolded. In *E. coli*, the periplasmic protein disulfide isomerase EcDsbC is responsible for the rearrangement of those incorrect disulfides, which is crucial for production of non-consecutive multi-disulfide-bonded proteins in the periplasm. EcDsbC is a 23.3 kDa protein that folds into a V-shaped homo-dimer (McCarthy et al., 2000). Each monomer of DsbC consists of two domains: a C-terminal catalytic domain adopting a thioredoxin fold and an N-terminal dimerization domain that also plays a role in substrate binding. The C- and N-terminal domains are connected via a long alpha-helical linker. The amino-terminal cysteine pairs, which are part of the active site CXXC motif of EcDsbC, are maintained in their reduced state by the inner membrane protein DsbD. EcDsbD, is responsible for maintaining the amino-terminal cysteine pairs of the EcDsbC in their reduced state. EcDsbD consisting of three domains catalyzes the transfer of electrons from the cytoplasm to the periplasm. The electrons flow from cytosolic NADPH via thioredoxin 1 (Trx1) and then via the β, γ, and α domains of DsbD to the EcDsbC (Katzen and Beckwith, 2000; Collet et al., 2003; Stirnimann et al., 2006a,b). Some microorganisms also possess the inner membrane protein CcdA, which is a functional homolog of DsbD and only consists of the β transmembrane domain of DsbD. In contrast to DsbD, which transfers reducing potential to a large numbers of extracytoplasmic proteins (Cho and Collet, 2013), CcdA is thought to be only involved in the cytochrome *c* maturation process (Katzen et al., 2002). However, recently published data, have shown that in *Bacillus subtilis, B. anthracis*, and *Streptococcus pneumoniae* CcdA plays a role also in sporulation and virulence (Erlendsson et al., 2004; Han and Wilson, 2013; Saleh et al., 2013).

An extensive bioinformatic screening for DsbA homologs, combines with recently carried out numerous functional and structural studies of DsbAs documented an enormous diversity of the pathways of disulfide bond formation within the bacterial kingdom. For instance, certain bacteria possess multiple DsbAs with different substrate specificities cooperating with one DsbB, while some others possess DsbA homologs but lack a homolog of DsbB. In this latter case, DsbA is reoxidized by a protein with DsbB-like activity and which is the bacterial homolog of the vitamin K epoxide reductase (VKOR; Li et al., 2010; Landeta et al., 2015). DsbA homologs from different organisms display very low overall sequence identity (15–40%) and various biochemical characteristics as reviewed by Shouldice et al. (2011) and McMahon et al. (2014). The comparative analysis of their structural and biochemical features allowed to recognize two main DsbAs classes (McMahon et al., 2014).

Recently published data, have shown that certain bacteria encode Dsb proteins involved in disulfide bond formation but fold into a V-shaped homodimeric molecule similar to EcDsbC. These bacteria include Gram–negative species, such as *Helicobacter pylori* or *Legionella pneumophila*, as well as Gram-positive organisms like *Corynebacterium diphteriae*. Even though, the dimeric DsbAs have been shown to play an important role in the correct folding of virulence factors, they still remain poorly characterized when compared to monomeric DsbAs. Preliminary data indicate that these dimeric enzymes that are capable of disulfide bond formation vary greatly in their catalytic motifs, structure and biochemical properties (Daniels et al., 2010; Roszczenko et al., 2012; Kpadeh et al., 2013).

HP0231 (DsbK) is a dimeric oxidoreductase that was previously described by our research group as a major oxidoreductase of *H. pylori* (Roszczenko et al., 2012) and recently characterized by Lester et al. (2015). Its structure has been solved by Yoon et al. (2011). HP0231 acts as periplasmic oxidase in *H. pylori* and *E. coli* cells, despite its structural resemblance to EcDsbG. However, its catalytic CXXC motif is identical to that of EcDsbA (i.e., CPHC) and differs from that of EcDsbC/G (i.e., CGYC/CPYC). The *cis*-Pro loop of HP0231, which is also necessary for disulfide bond formation, is V*c*P as in EcDsbA. Here we report a further detailed characterization of HP0231 by (1) conducting a phylogenetic analysis of HP0231, (2) exploring the functioning of HP0231 *in vivo* and *in vitro* and (3) analyzing the biochemical, functional and structural features of a truncated form of HP0231 lacking the dimerization domain (denoted HP0231m).

## Materials and Methods

### Bacterial Strains, Primers, Plasmids, Media, and Growth Conditions

Bacterial strains, plasmids and primers used in this study are listed in **Tables 1** and **2**. More extensive table of strains is included as Supplementary Table S1. *H. pylori* strains (26695 and N6) were grown on Blood Agar base no. 2 (BA) plates (Merck) supplemented with 10% (v/v) horse blood and Oxoid^TM^ *Helicobacter pylori* Selective Supplement (Dent) (ThermoScientific) or Mueller Hinton Broth (MH) supplemented with 10% (v/v) Fetal Bovine Serum (FBS; Lonza) at 37°C under microaerobic conditions provided by Anoxomat Mark II OP (MART Microbiology B.V) or CampyGen (ThermoScientific). For the selection of *H. pylori* N6 *hp0231::cat* complemented strains, kanamycin (25 μg ml^-1^) or/and chloramphenicol (10 μg ml^-1^) were added to the growth media. The *H. pylori* N6 *hp0231::cat* was employed for complementation experiments by HP0231 and HP0231m or EcDsbA. *E. coli* strains were grown at 37°C on solid or liquid Luria-Bertani (LB) medium or on M63 minimal medium (Hiniker et al., 2005). When needed, media were supplemented with antibiotics at the following concentrations: 100 μg ml^-1^ ampicillin, 30 μg ml^-1^ kanamycin, and 20 μg ml^-1^ chloramphenicol. The *E. coli* strains JCB816 (wt), JCB817 (*dsbA::kan*) and JCB818 (*dsbAB::kan*; Bardwell et al., 1991), PL263 (*mdoGdsbC::kan)*, PL284 (*mdoGdsbC::kan/*pBAD33*)*, PL285 (*mdoGdsbC::kan/dsbC^+^;* Leverrier et al., 2011) were employed for complementation experiments by HP0231 and HP0231m.

**Table 1:** Strains and plasmids used in this study.

Name	Relevant characteristics	Source
***H. pylori* strains**		
N6	*H. pylori* wild-type	Behrens et al., 2012
PR378	N6	Roszczenko et al., 2012
PR397	N6 pUWM397 (*hp0231^+^ in trans*)	Roszczenko et al., 2012
KBO570	N6 *hp0231::cat/pUWM570 (EcdsbA^+^ in trans* under native *hp0231* promoter)	This study
KBO574	N6 *hp0231::cat/pUWM574 (hp0231m^+^ in trans)*	This study
PR305	N6 *dsbI::aph*	Roszczenko et al., 2012
KBO571	N6 *dsbI::aph*/pUWM571 (*EcdsbA in trans* under native *hp0231* promoter)	This study
KBO575	N6 *dsbI::aph*/pUWM575 *(hp0231m^+^ in trans)*	This study
***Escherichia coli* strains**		
BL21/*EcdsbA^+^*	BL21 carrying pET28a/*EcdsbA*	JFC collection
BL21/*EcdsbC^+^*	BL21 carrying pET28a/*EcdsbC*	JFC collection
BL21/*EcdsbG^+^*	BL21 carrying pET28a/*EcdsbG*	JFC collection
KBO2030	Rosetta carrying pUWM591(*hp0231m^+^*)	This study
KBO2044	Rosetta carrying pUWM525 (*hp0231*)	This study
KBO519	JCB816 carrying pHel2	This study
PR501	JCB817 carrying pHel2	Roszczenko et al., 2012
PR521	JCB818 carrying pHel2	Roszczenko et al., 2012
KBO523	JCB819 carrying pHel2	This study
KBO520	JCB816 carrying pUWM500 (*HP0231^+^ in trans)*	This study
PR503	JCB817 carrying pUWM500 (*HP0231^+^ in trans)*	Roszczenko et al., 2012
PR522	JCB818 carrying pUWM500 (*HP0231^+^ in trans)*	Roszczenko et al., 2012
KBO524	JCB819 carrying pUWM500 (*HP0231^+^ in trans)*	This study
KBO576	JCB817 carrying pUWM575 (*HP0231m^+^ in trans)*	This study
KBO586	JCB818 carrying pUWM575 (*HP0231m^+^ in trans)*	This study
PL284	PL263 (*mdoGdsbC::kan*) carrying pBAD33	Leverrier et al., 2011
PL285	PL263 carrying JFC355 (*dsbC^+^ in trans*)	Leverrier et al., 2011
KBO2087	PL263 carrying pUWM500 (*HP0231^+^ in trans)*	This study
KBO2088	PL263 carrying pUWM575 (*HP0231m^+^ in trans)*	This study
**Plasmids**		
pUWM389	*hp0231^+^* in pGEM T-Easy	Roszczenko et al., 2012
pUWM397	*hp0231^+^* in pHel3	Roszczenko et al., 2012
pUWM500	*hp0231^+^* in pHel2	Roszczenko et al., 2012
pUWM574	*hp0231m^+^* in pHel3	This study
pUWM575	*hp0231m^+^* in pHel2	This study
pUWM570	*EcdsbA^+^* in fusion with promoter of *hp0231* gene in pHel3	This study
pUWM571	*EcdsbA^+^* in fusion with promoter of *hp0231* gene in pHel2	This study
pUWM525	*hp0231^+^* in pET28a	Roszczenko et al., 2012
pUWM2029	*hp0231m^+^* in pET28a	This study
pET28a/*EcdsbA*	*EcdsbA^+^* in pET28a	JFC collection
pET28a/*EcdsbC*	*EcdsbC^+^* in pET28a	JFC collection
pET28a/*EcdsbG*	*EcdsbG^+^* in pET28a	JFC collection

**Table 2:** Primers used in this study.

Name	Sequence 5′–3′	Orientation	Restriction site
HP231_BamL	GTGGGATCCGCCTGCTCTTCATCAATAACTTTAG	Forward	BamHI
HP231_XhoR	TACTCGAGCTTGTGGGGATTTGTAGGTC	Reverse	XhoI
HP231_katR	CAATTTCGCGCTATTTTGGTCATTAGCTGAAAC	Reverse	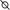
HP231_katL	GTTTCAGCTAATGACCAAAATAGCGCGAAATTG	Forward	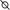
DsbA_231promL	GAACTTTAGGAGTTTTAATGAAAAAGATTTGGCTG	Forward	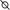
231prom_DsbAR	CAGCCAAATCTTTTTCATTAAAACTCCTAAAGTTC	Reverse	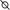
EcDsbA_R2	GTACTCGAGCGGCTAACGCAACAATAACAC	Reverse	XhoI
231expIa	GAGGCCATGGCTAATGACCAAAATAGCGCG	Forward	NcoI
231expII	GTGCTCGAGTGCCTTATAATGGTATAAGAA	Reverse	XhoI
HcpC_N6_XhoI_down	CGCTCGAGAACTTTGATTTTGAGCTGCTTGAGAATATCG	Reverse	XhoI
HcpC_N6_NcoI_up	GCACCCATGGCAGAGCAAGACCCTAAA	Forward	NcoI

### General DNA Manipulations

Standard DNA manipulations were carried out as described in the Sambrook manual (Sambrook and Russel, 2001) or according to the manufacturer’s instructions. Polymerase chain reactions (PCR) were performed with PrimeStar HS DNA Polymerase (Takara) under standard conditions according to the manufacturer’s instructions. Synthetic oligonucleotides synthesis and DNA sequencing were performed by Genomed S.A., Warsaw, Poland.

#### Construction of the Complementation Vector Carrying *hp0231m*

The vector carrying *hp0231m* was constructed by a two-step PCR method. Briefly, primers HP231_BamL and HP231_katR were used to amplify the DNA region encoding promoter region of the *hp0231* gene with a native signal sequence (SS) from the chromosome of *H. pylori* 26695. The catalytic domain of the *hp0231* gene, including the active motif CXXC, was amplified by HP231_katL and HP231_XhoR primers, also from the chromosome of *H. pylori* 26695. The HP231_katL and HP231_katR primers contained 5′ leader nucleotide sequences complementary to each other. Each PCR product was purified by a Gel-Out extraction kit (A&A Biotechnology). Next a mixture of two purified products (in equal amounts) was used as a template in a single PCR reaction, using the primers HP231_BamL and HP231_XhoR. Subsequently, the resulting PCR product was purified and cloned into pGEM-T Easy, generating pUWM568. Finally, using BamHI and XhoI restriction enzymes, the 1.5 kb DNA region encoding *hp0231m* was transferred into pHel2 and pHel3, generating pUWM575 and pUWM574, respectively. Correct construction of the resulting plasmids was verified by sequencing. Production of a proper protein was confirmed by Western-blot using anti-HP0231 serum.

#### Construction of the Complementation Vector Carrying *EcdsbA*

First, to test if the *EcdsbA* promoter sequences would be active in *H. pylori* cells, a derivative of the shuttle vector pHel3 coding native *EcdsbA* was constructed, but the expression of EcDsbA in *H. pylori* N6 *hp0231::cat* cells was undetectable (Western-blot). Therefore, *EcdsbA* was cloned under the promoter of the *hp0231* gene. Briefly, primers HP231_BamL and 231prom_DsbAR were used to amplify the DNA region encoding the promoter region of the *hp0231*gene from the chromosome of *H. pylori* 26695. DsbA_231promL and EcDsbA_R2 were used to amplify the region encoding the *EcdsbA* gene, including its own SS from the chromosome of *E. coli* TG1. The 231prom_DsbAR and DsbA_231promL primers contained 5′ leader nucleotide sequences complementary to each other. Each PCR product was purified by a Gel-Out extraction kit (A&A Biotechnology). Next, a mixture of the two purified products (in equal amounts) was used as a template in a single PCR reaction, using the primers HP231_BamL and EcDsbA_R2. Subsequently, the resulting PCR product was purified and cloned into pGEM-T Easy to generate pUWM569. Finally, using BamHI and XhoI restriction enzymes, the 1.3 kb DNA region encoding EcDsbA was transferred into pHel2 and pHel3, generating pUWM571 and pUWM570, respectively. Correct construction of the resulting plasmids was verified by sequencing. Production of the proper protein was confirmed by Western-blot, using anti-EcDsbA serum.

#### Natural Transformation of *H. pylori*

The naturally competent *H. pylori* N6 *hp0231::cat* and *H. pylori* N6 *HpdsbI*::*aph* (*hp0595*) were mixed with appropriate plasmid DNA and grown on BA plates supplemented with chloramphenicol or kanamycin as previously described (Roszczenko et al., 2012).

### Protein Analysis

#### Overexpression and Purification of HP0231 and HP0231m

HP0231 was overexpressed by autoinduction from an *E. coli* Rosetta/pUWM525 (KBO2044) strain and purified as previously described (Roszczenko et al., 2012). The HP0231m expression vector was constructed by amplifying the region encoding the mature catalytic domain of HP0231m (amino acids 119–265) with primers 231expIa and 231expII with chromosomal DNA of *H. pylori* 26695 used as a template. For cloning the insert into pET28a and to create the HP0231m-His_6_ recombinant protein, NcoI and XhoI restriction enzymes were used to yield plasmid pUWM591. For crystallization and biochemical experiments, the protein was expressed and purified from *E. coli* Rosetta harboring pUWM591 (KBO2030 strain). Expression was induced by 0.5 mM IPTG at OD_600_ ∼0.6. After 18 h in 18°C, cultures were centrifuged (4000 *g*) and the cell pellet was suspended in 50 mM sodium phosphate, pH 8.0, 300 mM NaCl, 10 mM imidazole. Cells were disrupted by ultrasonication. The cell lysate was centrifuged (8000 *g*) and the resulting supernatant was applied onto Bio-Scale Mini Profinity IMAC Cartridges (Bio-Rad) containing Ni-charged resin. The protein was eluted with an imidazole gradient, using the NGC chromatography system (Bio-Rad). To obtain higher purity, HP0231 and HP0231m proteins were next loaded onto ENrich^TM^ SEC 70 size exclusion columns (Bio-Rad) and eluted with PBS or 50 mM Tris pH 7.5, 150 mM NaCl, respectively. For biochemical tests, samples were dialyzed on desalting columns (Bio-Rad) against phosphate buffer, pH 7.0 (insulin test) or PBS buffer (oxidative and refolding tests of RNaseA) and concentrated as needed (Amicon^®^ Ultra-4, 10,000 NMWL; Millipore).

#### Overexpression and Purification of EcDsbA, EcDsbC, and EcDsbG

These three *E. coli* proteins were overexpressed from *E. coli* BL21 harboring pET28a/EcDsbA, EcDsbC or EcDsbG (JFC lab) by autoinduction (Studier, 2005) or IPTG induction (Roszczenko et al., 2012), and then purified by affinity chromatography and dialyzed against PBS or phosphate buffer, as described above for HP0231. All proteins were later used in biochemical experiments. EcDsbA was also used for rabbit immunization (Animal Facility, Faculty of Biology, University of Warsaw). The anti-EcDsbA rabbit serum was specific and recognized native EcDsbA, as verified by Western-blot analysis.

#### Overexpression and Purification of HcpC

The HcpC expression vector was constructed by amplifying the region encoding the mature catalytic domain of HcpC (amino acids 24–290) with primers HcpC_N6_XhoI_down and HcpC_N6_NcoI_up. Chromosomal DNA of *H. pylori* N6, was used as a template. For cloning the insert into pET39b and to create the EcDsbA-HcpC-His_6_ recombinant protein, NcoI and XhoI restriction enzymes were used to yield plasmid pUWM2021. The protein was expressed and purified from *E. coli* Rosetta harboring pUWM2021 (RG2022 strain), as described above for HP0231. Recombinant protein was used for rabbit immunization (Animal Facility, Faculty of Biology, University of Warsaw). The anti-HcpC rabbit serum was specific and recognized native HcpC, as verified by Western-blot analysis.

#### Preparation of Subcellular Fractions

Subcellular protein fractions were prepared from 48-h *H. pylori* cultures. Periplasmic proteins were released from the cells using an osmotic-shock procedure (Myers and Kelly, 2005). After decanting the periplasmic fraction, bacterial pellets were resuspended in 20 mM Tris-HCl, pH 7.5 and sonicated to release the cell contents. Subsequently, cell wall debris was removed and the supernatants were ultracentrifuged (100 000 *g*, 4°C, 30 min) to separate the membrane and cytoplasmic fractions. Finally, the cell envelope was fractionated into inner and outer membranes by selective solubilization of the inner membrane with 2.0% (w/v) sodium lauryl sarcosine (Baik et al., 2004).

### Biochemical Assays

#### Determination of the *in vivo* Redox State of Proteins

The redox states of EcDsbA, HP0231, HP0231m, and HcpC were visualized by alkylating the free cysteine residues using 4-acetamido-4′-maleimidylstilbene-2,2′-disulfonic acid (AMS, Invitrogen) as described previously (Roszczenko et al., 2012; Denoncin et al., 2013).

#### Alkaline phosphatase (AP) assay

The ability of HP0231m to restore the activity of AP *in vivo* in *E. coli* cells was determined in minimal medium M63 as previously described (Roszczenko et al., 2012).

#### Insulin Reduction Assay

Reductase activity was assessed by an insulin precipitation assay (Bardwell et al., 1991; Kpadeh et al., 2013) using human insulin solution (Sigma). Reactions (triplicate) were carried out in 200 μl of 100 mM sodium phosphate buffer, pH 7.0, 131 μM insulin, 1 mM dithiothreitol (DTT), 2 mM EDTA, and 10 μM HP0231m or EcDsbA; reaction mixtures were incubated in a 96-well plate format at room temperature in a Sunrise^TM^ (Tecan) plate reader. Reactions were started by adding DTT to a final concentration of 1 mM. The changes in the absorbance (A_650_) as a function of time were measured (Collet et al., 2003; Kpadeh et al., 2013). The results are presented as the average of three independent experiments.

#### Chaperone Activity of HP0231 and HP0231m

The chaperone activity of HP0231 and HP0231m, in comparison to EcDsbG, was determined as described previously (Shao et al., 2000) using thermal aggregation of citrate synthase – CS (Sigma) and luciferase – LUC (Sigma) as chaperone substrate proteins. Briefly, reactions (triplicate) were carried out in 2 ml of 40 mM HEPES, pH 7.5, 0.15 μM CS or 0.4 μM LUC and 0.15 μM (for CS) or 0.2 μM (for LUC) HP0231 or 0.2 μM HP0231m, or with 0.2 μM EcDsbG as a positive control and BSA as a negative control, all at 43°C. Protein aggregation was monitored with light scattering measurements, using a Varian spectrofluorometer. The excitation and emission wavelengths were set to 350 nm for measurements when LUC served as a substrate protein and to 500 nm when CS served as a substrate protein. The excitation and emission slit widths were set to 2.5 nm. Three independent experiments were performed.

#### Determination of the Redox Potential of HP0231m

The redox potential of HP0231m was fluorometrically determined from the equilibrium constant with glutathione, as previously described (Roszczenko et al., 2012). The results are presented as the average of three independent experiments.

#### Oxidative Folding of Reduced RNaseA

*In vitro* oxidative folding of reduced RNaseA was performed with HP0231, HP0231m, and EcDsbA as described earlier, with a few modifications (Daniels et al., 2010; Chim et al., 2013). Proteins were oxidized with 50 mM oxidized glutathione (GSSG) and incubated for 1 h at room temperature. RNaseA was reduced by overnight incubation at room temperature in 100 mM Tris acetate pH 8.0, containing 6 M guanidine hydrochloride and 140 mM DTT. All proteins were then dialyzed on desalting columns (Bio-Rad) and concentrated in PBS. Native RNaseA and EcDsbA were used as positive controls. The redox state of the thiols was confirmed by the Ellman’s assay, which exploits the colorimetric change at A_412_ when 5,5′-dithio-bis-(2-nitrobenzoic acid) (DTNB; ThermoScientific) is converted to 2-nitro-5 thiobenzoate (TNB) upon cleavage of the disulfide bond by free thiols. Oxidase activity was measured by analyzing the cleavage of cCMP (Sigma; cytidine 2′:3′-cyclic monophosphate monosodium salt) at A_296_ by refolded RNaseA in the presence of tested enzymes. Reactions (triplicate) were carried out in 200 μl of PBS buffer containing 100 mM Tris acetate pH 8.0, 2 mM EDTA, 0.2 mM GSSG, 1 mM GSH, 4.5 mM cCMP, RNaseA (10 μM) and analyzed enzyme (20 μM). The reaction mixtures were prepared in a 96-well plate format and read through 30 min at 27°C in a Sunrise^TM^ (Tecan) plate reader. Three independent experiments were performed.

#### Refolding of Scrambled RNaseA (scRNase)

*In vitro* refolding of scrambled RNaseA was performed for HP0231, HP0231m, and EcDsbC as described earlier, with a few modifications (Messens et al., 2007; Daniels et al., 2010; Chim et al., 2013). Proteins were reduced with 100 mM DTT and incubated overnight at 4°C. RNaseA was first reduced by overnight incubation at room temperature in 100 mM Tris acetate pH 8.0, containing 6 M guanidine hydrochloride and 140 mM DTT. Then, in order to introduce incorrect disulfides, the reduced RNaseA was dialyzed against PBS buffer containing 6 M guanidine hydrochloride, sparged with oxygen and incubated for 3 days in the dark at room temperature. Finally, 2 mM hydrogen peroxide (Sigma) was added for 30 min at 25°C. All proteins were then dialyzed on desalting columns (Bio-Rad) and concentrated in PBS. Native RNaseA and EcDsbC were used as a positive control. The redox state of the thiols was confirmed by the Ellman’s assay. RNaseA activity was measured by analyzing the cleavage of cCMP, as described above for the oxidative test, but with the reaction mixture changed to 100 mM Tris acetate pH 8.0, 2 mM EDTA, 10 μM DTT, 4.5 mM cCMP, RNaseA (40 μM) and analyzed enzyme (20 μM). Three independent experiments were performed.

### Phenotype Assays

#### Spot Titers for Cadmium Resistance

Spot titers for cadmium resistance were performed to quantify the relative oxidase activity of HP0231m *in vivo* (Ren et al., 2009). Briefly, *H. pylori* cells were harvested from BA plates after 24 or 48 h of incubation under microaerobic conditions. Samples were standardized using OD_600nm_ of the culture and serially diluted with 150 mM NaCl. Then, 4 μl of each dilution was plated onto BA plates, supplemented with 8–12 μM cadmium chloride. *H. pylori* cells were incubated 5–7 days under microaerobic conditions. All spot titers were performed in triplicate.

#### Motility Assays

*Helicobacter pylori* cells were harvested from BA plates after 24 or 48 h of incubation under microaerobic conditions. Samples were standardized using OD_600nm_ of the culture. Next, cells were inoculated with a sterile toothpick onto Mueller-Hinton (MH) soft agar plates containing 0.35% (w/v) agar and 10% (v/v) FCS, and incubated for 4–5 days at 37°C under microaerobic conditions. *E. coli* cells were grown in liquid culture in LB broth until the OD_600nm_ value was 0.6. Then bacteria were inoculated with a sterile toothpick onto LB soft agar plates containing 0.35% (w/v) agar and incubated overnight at 37°C. All motility assays were performed in triplicate.

### Bioinformatics Analyses

In proteins HP0231, LpDsbA2, EcDsbG, EcDsbA, Cj1380, CG_0026, and CG_2799, the sequences corresponding to the thioredoxin catalytic domains were defined using an on-line version of HHpred (Biegert et al., 2006). These sequences were subsequently used as queries for PSI-BLAST searches (three iterations) on nr90 database (NCBI non-redundant protein sequence database filtered at 90% sequence identity). The matching sequences obtained from the individual PSI-BLAST runs were grouped together, and redundant, poorly matching (*e*-value > 1^e-3^) and truncated (query coverage < 90%) sequences were removed. The final set of 6181 sequences was clustered according to the pair-wise sequence similarity using CLANS (Frickey and Lupas, 2004; *p*-value cut-off 1^e-11^) and the clusters corresponding to HP0231, DsbG, DsbC, and class I and class II DsbA were defined manually. Additionally, a sub-cluster of the class II DsbA cluster, containing the sequence of the catalytic domain of the LpDsbA2 protein, was defined. Multiple sequence alignments of sequences contained in the individual clusters were built with MUSCLE (Edgar, 2004) and used to calculate sequence logos with WebLogo (Crooks et al., 2004).

For each of 6181 catalytic domain sequences, an upstream region (i.e., from the N-terminal end of a protein to the beginning of the catalytic domain) was extracted. After removing sequences shorter than 50 amino acids, the remaining 3304 sequences were searched using a local version of HHpred (Remmert et al., 2012) and a database of sequence profiles corresponding to the dimerization domains of DsbG/C and HP0231. Moreover, the sequences were scanned for the presence of coiled-coil domain motifs, using MARCOIL (Delorenzi and Speed, 2002) and PCOILS (Lupas et al., 1991). Finally, the sequences were clustered with CLANS (*p*-value cut-off 5^e-6^) and two clusters were defined (see **Figure 2**).

## Results

### Phylogenetic Analysis of HP0231

**FIGURE 1:**
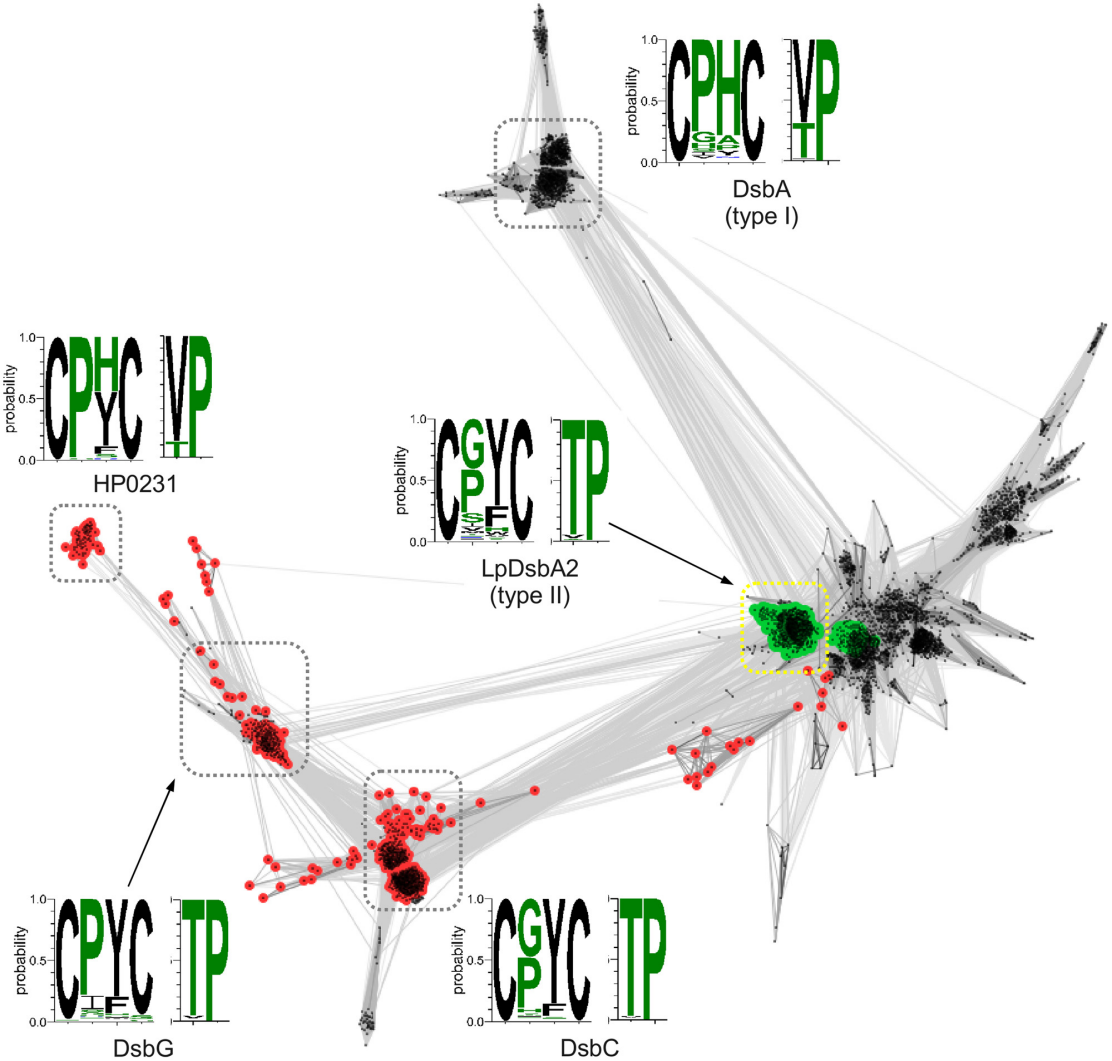
**Clustering of Dsb catalytic domain sequences.** Points and edges correspond to Dsb catalytic domain sequences and their pair-wise similarities, respectively. Catalytic domains that contain an upstream dimerization domain (DsbG/C/HP0231-like – red and LpDsbA2-like – green) are indicated. Clusters of sequences are indicated with dashed boxes and for each cluster CxxX and X*c*P motifs conservation is shown as sequence logo in which height of a letter corresponds to the frequency of occurrence of a given amino acid.

To gain insight into the *H. pylori* Dsb system and the evolutionary origins of HP0231, we first decided to use a phylogenetic approach by performing sequence clustering analysis of the thioredoxin catalytic domains of Dsb proteins (see Materials and Methods for details; Kpadeh et al., 2013, 2015). The resulting map (**Figure 1**) revealed two clusters corresponding to type I and type II DsbA domains, as defined by McMahon et al. (2014). The type-II DsbA cluster is connected to the DsbC cluster and closely related to the DsbG cluster. The C-terminal catalytic domain of the HP0231 protein is localized in a tiny separate cluster, which is specifically connected to the DsbG cluster, whereas the catalytic domain of LpDsbA2, dimeric Dsb bifunctional protein of *L. pneumophila*, is localized in the DsbA type-II cluster. The closest homologs of HP0231 typically contain the CPHC/V*c*P motif, which is identical to the one observed in canonical DsbA proteins. The catalytic domains of the DsbG and DsbC proteins, despite being closely related to the catalytic domain of HP0231, typically contain CPYC/T*c*P and CGYC/T*c*P motifs, respectively. The most common motif for LpDsbA2 (*L. pneumophila* DsbA2 protein) homologs is CGYC/T*c*P.

**FIGURE 2:**
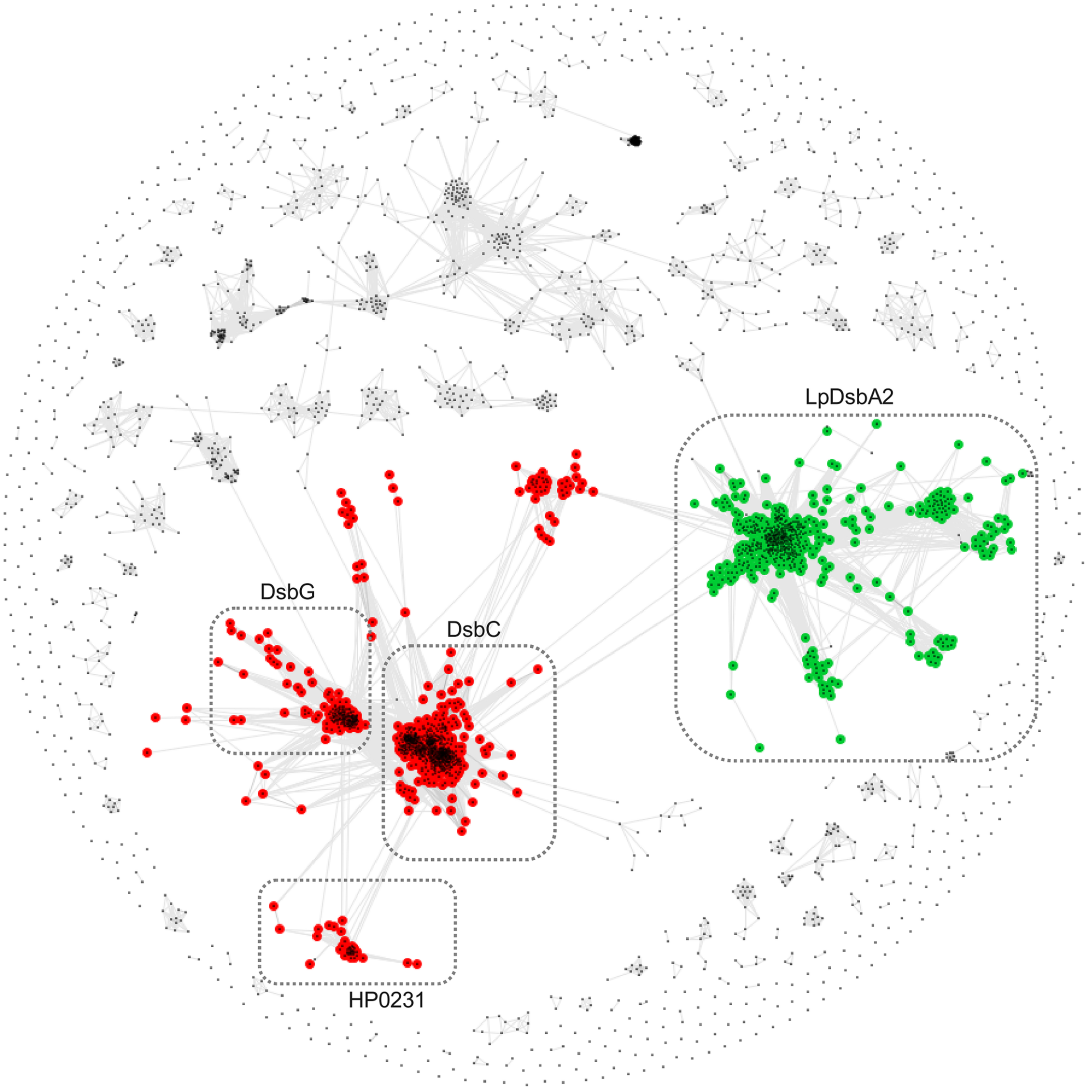
**Clustering of dimerization domain sequences.** Points and edges correspond to dimerization domain sequences and their pair-wise similarities, respectively. Dimerization domains that are located upstream DsbG/C/HP0231-like and LpDsbA2-like catalytic domains are shown in red and green, respectively. Clusters of sequences are indicated with dashed boxes and they correspond to clusters defined in the Dsb catalytic domains cluster map (see **Figure 1**).

In addition to the clustering of the catalytic C-terminal domains, we performed an analogous analysis with the N-terminal domain of HP0231. To this end, we extracted sequences preceding the catalytic domains of Dsb proteins, as shown in **Figure 1**, and clustered them with CLANS, a method that displays the pair-wise sequence similarities measured as BLAST *p*-values in form of two-dimensional map. The resulting map (**Figure 2**) revealed two large clusters; the first contained sequences homologous to the N-terminal dimerization domains of DsbG/C proteins, and the second encompassed homologs of the N-terminal domain of *L. pneumophila* DsbA2. The sequences from the two clusters share no homology and apparently evolved independently. Analysis of the LpDsbA2 N-terminal domain sequence and its homologs revealed a putative coiled-coil domain, probably (*e*-value: 0.022) related to the zinc resistance-associated protein (pdb: 3lay). The HP0231 dimerization domain is localized in a separate cluster related to the DsbG/C dimerization domains. The sequences on the catalytic domain cluster map (**Figure 1**) were colored according to the presence of either the DsbG/C/HP0231-like dimerization domain (red) or the LpDsbA2-like dimerization domain (green).

### Construction of an HP0231 Derivative Lacking the Dimerization Domain (HP0231m) and Analysis of its Oxidoreductase and Isomerization Activities *in vivo*

Our recent work (using biochemical and genetic approaches) led to the characterization of the first dimeric oxidoreductase (HP0231) functioning in an oxidizing pathway in *H. pylori.* We also showed that it complemented the lack of DsbA in *E. coli* when delivered on a low-copy plasmid (Roszczenko et al., 2012).

**FIGURE 3:**
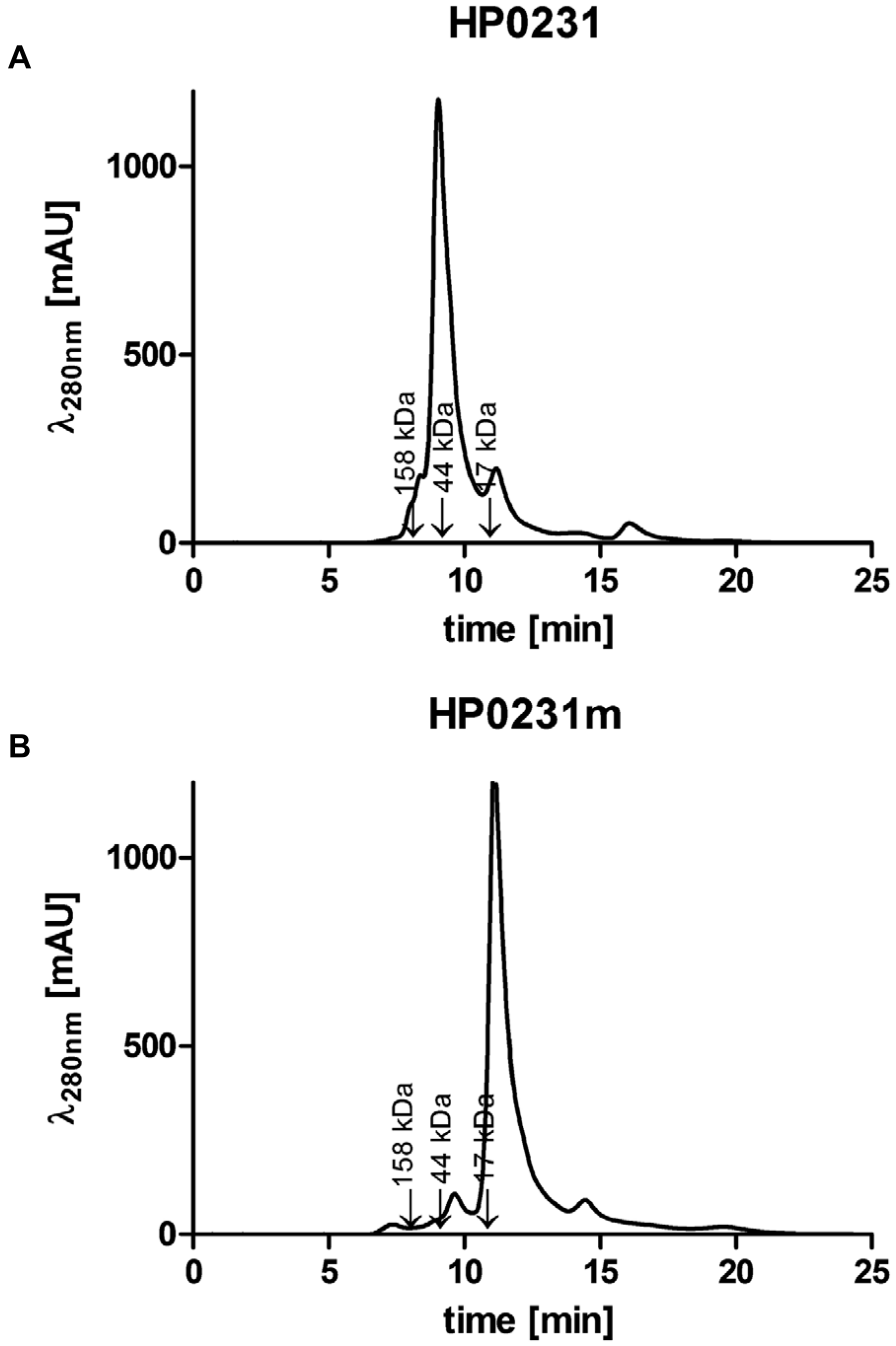
**Size exclusion profiles of the purified *Helicobacter pylori* proteins separated on an ENrich^TM^ SEC70 column (Bio-Rad) and monitored by absorbance at 280 nm. (A)** HP0231 elutes as a single peak at 9.05 min, with an estimated mass of 56 kDa, consistent with the size of the homodimer. **(B)** HP0231m elutes at 11.1 min, with estimated mass of 18 kDa, consistent with the size of the monomer. The column was calibrated with Gel Filtration Standard (Bio-Rad): Thyroglobulin (670 kDa), γ-globulin (158 kDa), Ovalbumin (44 kDa), Myoglobin (17 kDa), Vitamin B12 (1.35 kDa). The relative positions of the chosen standards are marked with arrows.

**FIGURE 4:**
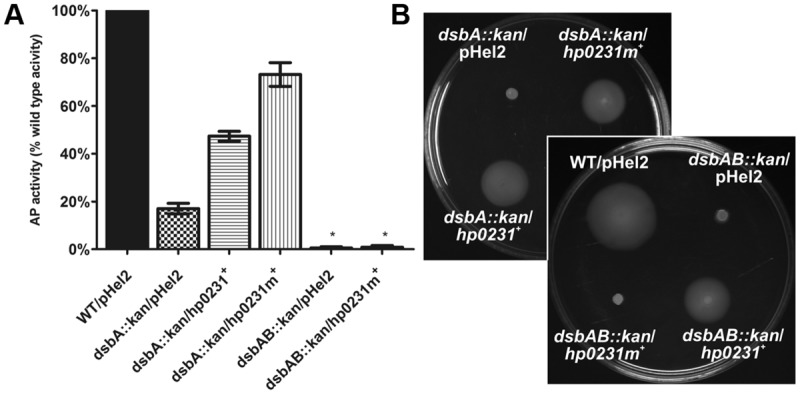
**HP0231m restores the *Escherichia coli dsbA::kan* wild type phenotype in a DsbB-dependent manner in two functional assays.** As negative controls, *E. coli dsbA::kan* and *E. coli dsbAB::kan* were transformed with an empty pHel2 vector. **(A)** Alkaline phosphatase (AP) assay. The bars represent average activity of three independent experiments (*n* = 3) with the wild type set to 100% activity. There are significant differences (*p* < 0.001) in relative AP activity between the *E. coli wt* cells and the *E. coli dsbA::kan* and *E. coli dsbAB::kan* mutant strains, and also the strains complemented *in trans* by *hp0231* and *hp0231m*. Error bars marked with asterisk (^∗^) indicate no significant difference between *dsbAB::kan*/pHel2 and *dsbAB::kan/hp0231m^+^* (ANOVA followed by *post hoc* Tukey’s test). AP activity of wild type and *dsb* mutants and complemented strains was performed in M63 minimal medium. **(B)** Motility of the *E. coli dsbA::kan* and *E. coli dsbAB::kan* complemented *in trans* by *hp0231* and *hp0231m*. Bacterial motility was monitored after 24 h of incubation on 0.35% LB-agar plates. The *E. coli dsbAB::kan/hp0231m^+^* is non-motile while *E. coli dsbA::kan/hp0231m^+^* and *E. coli dsbAB::kan/hp0231^+^* are motile. The figure presents a representative result.

**FIGURE 5:**
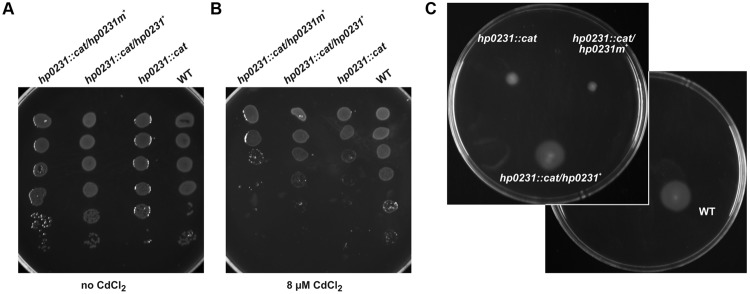
**HP0231m is not active in *H. pylori hp0231::cat* cells.** As a positive control, *H. pylori* N6 *hp0231::cat* was transformed with pHel2 carrying the native *hp0231* gene. **(A,B)** cadmium sensitivity assay. Exponentially growing *H. pylori* (wt, the *hp0231::cat* mutant and *hp0231::cat* complemented *in trans* with *hp0231* or *hp0231m).* Cultures were 10-fold serially diluted, spotted on BA plates without **(A)** or with **(B)** 8 μM CdCl_2_, and incubated at 37°C. The mutant shows reduced growth after 3 days of incubation on plates containing cadmium chloride. HP0231, but not HP0231m, partially restore the cadmium resistance of the *H. pylori hp0231::cat*. **(C)** Motility assay. Bacterial motility was monitored after 4 days of incubation on 0.35% MH-agar plates containing 10% FCS. Both the *hp0231::cat* mutated strain and the same strain complemented *in trans* with *hp0231m* are non-motile.

To better understand functioning of the *H. pylori* Dsb network, we further examined the activity of HP0231 by performing various *in vivo* and *in vitro* experiments. Moreover, to assess the significance of its dimeric structure, we generated a truncated HP0231 lacking the dimerization domain, denoted HP0231m. The truncated protein was constructed by cloning part of the *hp0231* gene encoding a catalytic domain in frame with its native SS, under the control of an original promoter. For the complementation assays in *H. pylori hp0231^-^* or in *E. coli dsbA^-^/dsbAB^-^* mutants, the DNA fragment encoding HP0231m was cloned into either pHel3 (pUWM2017) or pHel2 (pUWM2014), respectively (Heuermann and Haas, 1998). We show in Supplementary Figure S1 how the different plasmids were constructed. Production of the monomeric version of HP0231 in *H. pylori*, as well as in *E. coli* cells, was confirmed by Western-blot analysis, using specific rabbit anti-HP0231 serum. To verify that HP0231m indeed exists as a monomeric protein, size exclusion chromatography was employed (**Figures 3A,B**). His_6_-tagged HP0231 and His_6_-tagged HP0231m purified from *E. coli* cells were applied to calibrated gel filtration column. HP0231 was eluted as a single peak at 9.05 min, corresponding to an approximate mass of 56 kDa, which is consistent with its dimeric structure. In contrast, HP0231m eluted at 11.1 min, with an approximate mass of 18 kDa, corresponding to a monomeric structure. We first tested whether truncated HP0231m can complement lack of DsbA in *E. coli*. Complementation tests showed that HP0231m was able to restore both motility and AP activity (**Figures 4A,B**), as observed with native EcDsbA. Moreover, the HP0231m activity was fully EcDsbB-dependent, as judged by its inability to allow bacterial motility or regenerate AP activity in a double mutated *E. coli dsbAB* strain. Note that we previously showed that complementation by dimeric HP0231 does not require DsbB (Roszczenko et al., 2012). In contrast to what was observed in *E. coli* cells, complementation tests in *H. pylori* revealed that HP0231m was not able to restore motility or cadmium resistance – two phenotypes that are defective in *H. pylori hp0231^-^* cells (**Figures 5A–C**). Also EcDsbA, expressed from the native *hp0231* promoter (pUWM570), was not active when introduced into *H. pylori hp0231^-^*, as measured by the same tests Supplementary Figure S2.

In order to introduce disulfide bonds, DsbA proteins need to be regenerated by a protein like DsbB that transfers the electrons to the respiratory chain (Inaba, 2008; Inaba and Ito, 2008). It is not completely clear how HP0231 is re-oxidized *in vivo*. *H. pylori* does not encode a classical DsbB, although it does encode a DsbB-like protein – HpDsbI (HP0595). As previously shown, mutation of the *hpdsbI* gene produces HP0231 in the reduced form, though the active, oxidized form remains more pronounced (Roszczenko et al., 2012). Thus we decided to check whether the HP0231 in cells that lack HpDsbI is still active. To do this, we performed a motility test for *dsbI* mutated cells, which showed that partially oxidized HP0231 still assures cell movement Supplementary Figure S3. These results suggest that HpDsbI plays a role in HP0231 reoxidation but other mechanisms may also be involved.

**FIGURE 6:**
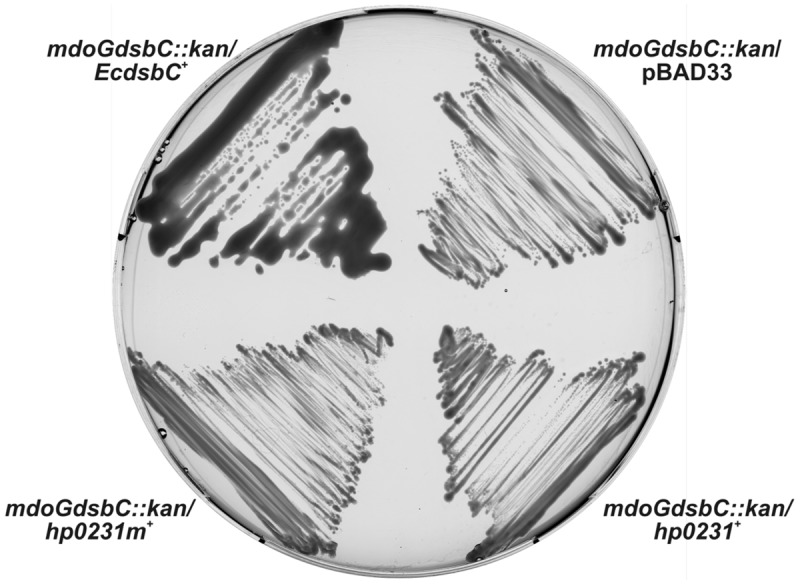
**HP0231 and HP0231m cannot function as a reductase in an *mdoGdsbC::kan* strain.** The *E. coli mdoGdsbC::kan* strain harboring various recombinant plasmids (pBAD33, pBAD33/*dsbC^+^*, pHel2/*hp0231^+^*, pHel2/*hp0231m^+^*) were grown on M63 minimal medium for 2 days at room temperature. The mucoid phenotype of the *mdoGdsbC/dsbC^+^* strain was observed. Neither *hp0231* nor *hp0231m* complements the *dsbC* mutation.

*Helicobacter pylori* proteome does not contain the classical homodimeric DsbC, which in many Gram-negative bacteria rearranges incorrectly paired cysteines (McCarthy et al., 2000). Previously we showed that HP0231 does not complement an *EcdsbC* mutant as assessed by copper sensitivity (Roszczenko et al., 2012). Copper is known to catalyze the formation of non-native disulfide bonds in many periplasmic proteins, and therefore, this assay measures the global effect of EcDsbC activity (Hiniker et al., 2005). In order to confirm that result in a more direct manner, we decided to examine HP0231 isomerization activity by checking its influence on the structure of a specific protein, EcRcsF. EcRcsF is a small outer-membrane lipoprotein which activates the Rcs phosphorelay upon envelope stress, and contains two non-consecutive disulfide bonds (Cho et al., 2014). One of them is necessary for proper EcRcsF folding, and the process of disulfide bond formation is EcDsbC dependent. Mutation of the *mdoG* gene, involved in the synthesis of membrane-derived oligosaccharide, activates the Rcs system in an RcsF-dependent manner. An *mdoG* mutant displays a mucoid phenotype on M63 minimal medium, while a double *mdoGdsbC* mutant does not, due to lack of activation of the Rcs cascade (Leverrier et al., 2011). We examined the ability of HP0231 and HP0231m to complement the deficiency of DsbC in an *mdoGdsbC* double *E. coli* mutant. We found that neither HP0231 nor HP0231m was capable of complementing the lack of EcDsbC, further suggesting that HP0231 cannot function as an isomerase in a heterologous host, *E. coli* (**Figure 6**).

### Redox State of HP0231m and HP0231 in *E. coli* and *H. pylori* Cells

**FIGURE 7:**
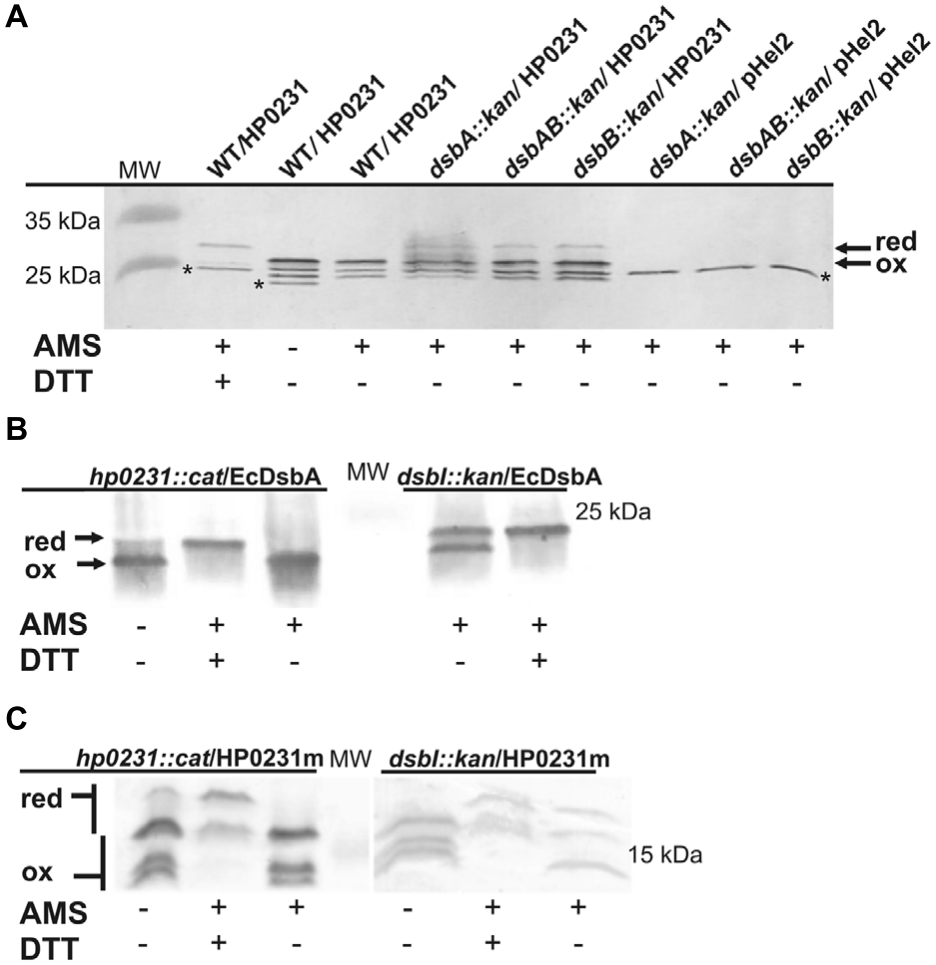
**Redox state of HP0231 in *E. coli* and HP0231m and EcDsbA in *H. pylori*. (A)** HP0231 in *E. coli* wt, *E. coli dsbA::kan* and *E.coli dsbAB::kan* strains. Asterisk (^∗^) mark unspecific bands. **(B,C)** EcDsbA **(B)** and HP0231m **(C)** in *H. pylori* N6 *dsbI::kan* or *hp0231::cat* mutants. Bacterial cultures were treated with 10% TCA, followed by alkylation with AMS. Cellular proteins including the reduced (red; DTT treated, modified with AMS) and the oxidized (ox; non-modified with AMS) controls were separated by 18% SDS-PAGE under non-reducing conditions, followed by Western-blot analysis using rabbit antibodies against HP0231 or EcDsbA. Each lane contains proteins isolated from the same amount of bacteria.

HP0231 is present in *H. pylori* in an oxidized form, which is consistent with its function (Roszczenko et al., 2012). Considering that it does also act as an oxidase in *E. coli*, we next determined its redox state in *E. coli* cells. An AMS trapping experiment showed that HP0231 is present mainly in the oxidized form, as expected (**Figure 7A**). A small amount of HP0231 in the reduced form was detectable, independently of the genetic background of the host (*E. coli dsbA^-^* vs. *E. coli dsbAB^-^*). The extra immunoreactive proteins migrating faster than the oxidized form of HP0231 are *E. coli* protein/s reacting with anti-HP0231 serum, which also are modified by AMS. As the activity of HP0231, previously measured by the AP assay, was also EcDsbB independent, the mechanism of HP0231 reoxidation in both the native and the heterologous host still remains unexplained. The dimeric structure of HP0231 appears to be a structural barrier for its potential upstream redox partner when expressed in *E. coli*, as monomeric HP0231m interacts with EcDsbB whereas dimeric HP0231 does not.

Next, the redox state of HP0231m in *H. pylori* cells was determined using the AMS trapping strategy and specific rabbit anti-HP0231 serum. We found that HP0231m was present predominantly in the oxidized form, the absence of HpDsbI resulting only in the appearance of small amounts of the proteins in the reduced forms. Also EcDsbA is present in *H. pylori* in the oxidized form (**Figures 7B,C** – the presence of HP0231m in three forms makes interpretation difficult). Consequently, based on *in vivo* experiments, we concluded that the inability of HP0231m to complement the lack of native HP0231 is probably due to the lack of dimeric structure.

### Biochemical Properties of HP0231m Compared to Native Dimeric HP0231

In order to further characterize HP0231m, we analyzed its biochemical properties in comparison to HP0231. The recombinant proteins used for biochemical analysis were produced as cytoplasmic proteins in *E. coli* cells and purified by affinity chromatography. First, to gain insight into the mechanism of HP0231m action, we determined its redox potential by equilibrating this protein in a series of redox buffers containing various ratios of reduced and oxidized glutathione. We found that the HP0231m redox potential was -112 mV Supplementary Figure S4, identical to that previously determined for HP0231 and similar to that of EcDsbA (Inaba and Ito, 2002; Roszczenko et al., 2012).

**FIGURE 8:**
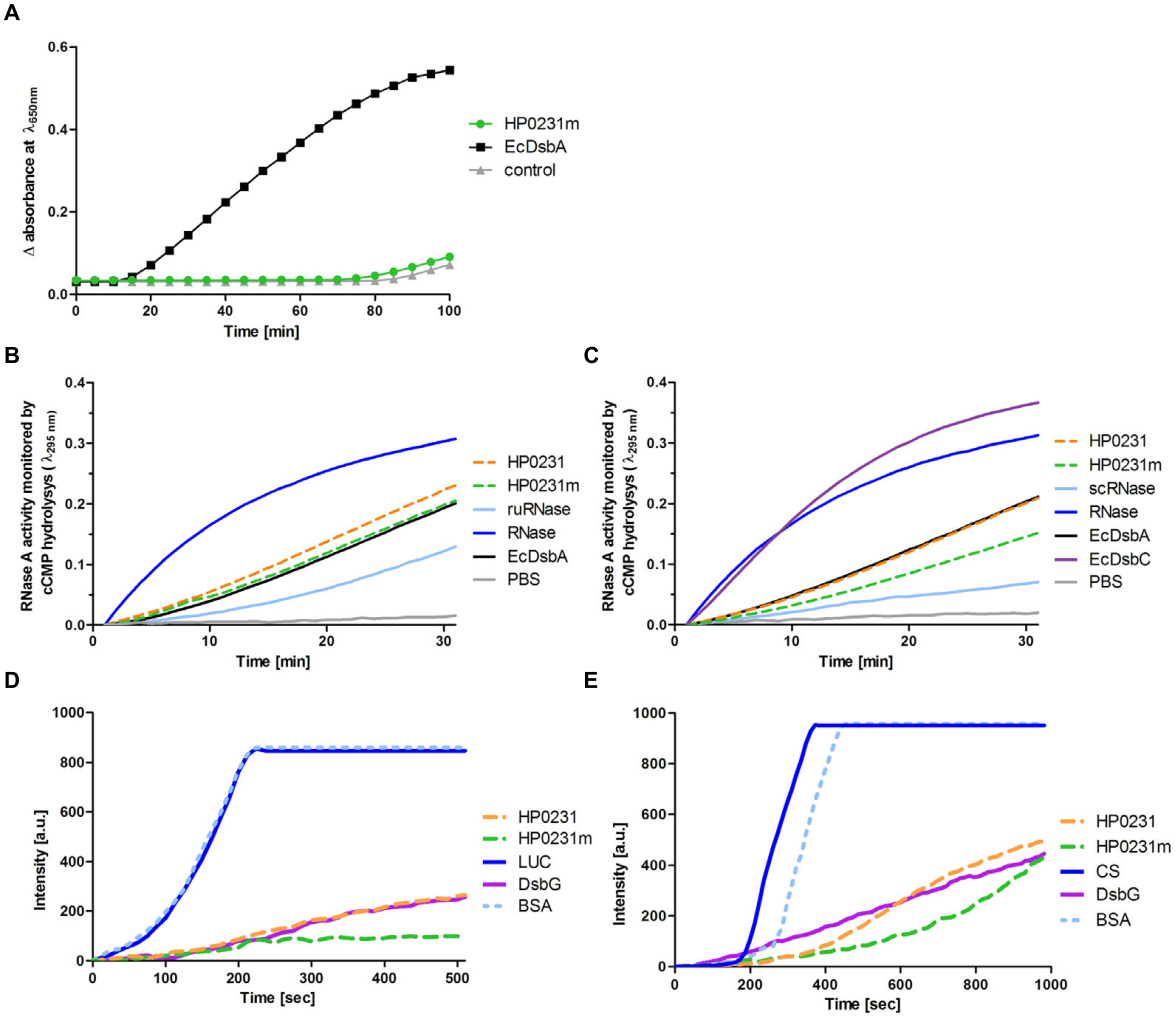
**Biochemical assays performed on purified HP0231 and HP0231m.** Purified EcDsbA, EcDsbC, or EcDsbG were used as controls. **(A)** HP0231m is not active in the insulin reduction assay. The reaction contained 131 μM insulin in potassium phosphate buffer, pH 7.0 and 2 mM EDTA. The reaction was performed in the absence or presence of 10 μM EcDsbA and 10 μM HP0231m. Reactions were started by adding DTT to the final concentration of 1 mM. Changes in the absorbance at 650 nm as a function of time were measured. The figure presents the average of three independent experiments (*n* = 3). **(B)** HP0231 and HP0231m are active in an oxidase activity assay (reduced unfolded – ruRNase activity assay). Reactions were carried out in 200 μl of PBS buffer containing 100 mM Tris acetate pH 8.0, 2 mM EDTA, 0.2 mM GSSG, 1 mM GSH, 4,5 mM cCMP, ruRNaseA (10 μM) and the analyzed enzyme (20 μM). The reaction was performed in the absence or presence of 20 μM EcDsbA, 20 μM HP0231, or 20 μM HP0231m. Changes in absorbance at 296 nm as a function of time were measured. Three independent experiments were performed. The figure presents a representative result. **(C)** HP0231 and HP0231m cannot work as isomerases in the scrambled RNase (scRNase) activity assay. Reactions were carried out in 200 μl of PBS buffer containing 100 mM Tris acetate pH 8.0, 2 mM EDTA, 10 μM DTT, 4.5 mM cCMP, scRNaseA (40 μM) and the analyzed enzyme (20 μM). Reactions were performed in the absence or presence of 20 μM EcDsbC, 20 μM HP0231, or 20 μM HP0231m. Changes in absorbance at 296 nm as a function of time were measured. Three independent experiments were performed. The figure presents a representative result. **(D)** HP0231 and HP0231m suppress the thermal aggregation of luciferase (LUC) in the chaperone activity assay. LUC was diluted to a final concentration of 0.10 μM into 40 mM HEPES-KOH buffer, pH 7.5, equilibrated at 43°C in the absence or in the presence of 0.15 μM HP0231 or 0.15 μM HP0231m, respectively. Protein aggregation was monitored with light scattering measurements using a Varian spectrofluorometer. The excitation and emission wavelengths were set to 350 nm. The excitation and emission slit widths were set to 2.5 nm. To exclude unspecific protein effects, control experiments in the presence of 1.5 μM bovine serum albumin were conducted. Three independent experiments were performed. The figure presents a representative result. **(E)** HP0231 and HP0231m suppress the thermal aggregation of citrate synthase (CS) at 43°C. 30 μM CS was diluted 200-fold into prewarmed 40 mM HEPES-KOH, pH 7.5, at 43°C in the absence or presence of 0.15 μM HP0231 and 1.5 μM HP0231m, respectively. Protein aggregation was monitored with light scattering measurements using a Varian spectrofluorometer. The excitation and emission wavelengths were set to 350 nm. The excitation and emission slit widths were set to 2.5 nm. To exclude non-specific protein effects, control experiments in the presence of 1.5 μM bovine serum albumin were conducted. Three independent experiments were performed. The figure presents a representative result.

Next, we evaluated the ability of HP0231m to reduce insulin in the presence of DTT, the assay commonly used to determine whether a protein functions as an oxidoreductase. Unexpectedly, although HP0231m complements the lack of EcDsbA *in vivo* and its redox potential is similar to that of EcDsbA, it was not active in this assay (**Figure 8A**). However, it should be noted that HP0231 catalyzes the insulin reduction even more efficiently than EcDsbA (Roszczenko et al., 2012).

We then decided to examine how efficiently they oxidize a protein a substrate *in vitro* using unfolded RNaseA as substrate (**Figure 8B**). We found that all proteins tested (EcDsbA, HP0231, and HP0231m) reactivated reduced unfolded RNase (ruRNase) at identical levels. We also examined the isomerization activity of both *H. pylori* proteins using scrambled RNase (scRNase) as a substrate and *E. coli* DsbC as a positive control. Neither HP0231 nor HP0231m was active in this test (**Figure 8C**).

**FIGURE 9:**
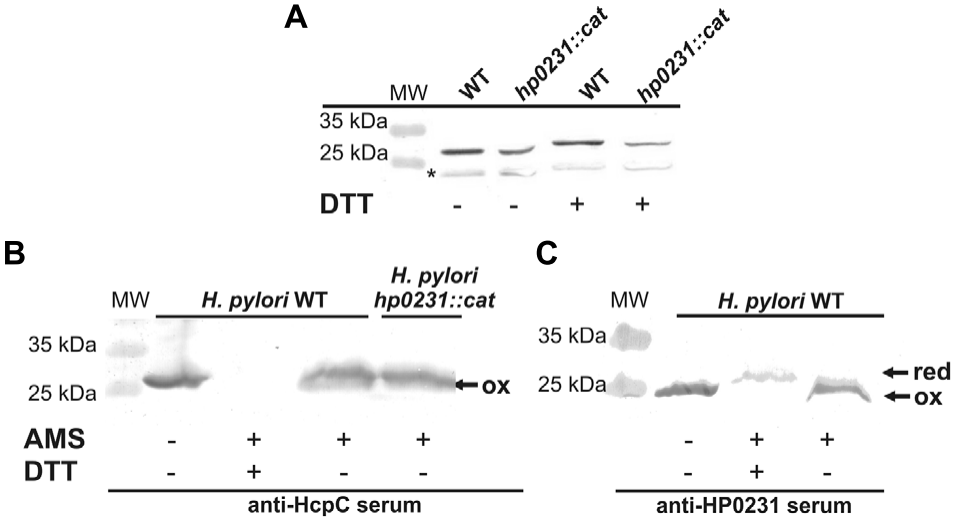
**Interaction between HP0231 and HcpC. (A)** Quantitative Western-blot analysis of HcpC in *H. pylori* wt and *H. pylori hp0231::cat* cells. Each lane contains the same amount of proteins. Samples (native or DTT treated) were separated by 12% SDS-PAGE under non-reducing conditions, followed by Western-blot analysis using specific rabbit antibodies against HcpC. **(B,C)** Redox state of HP0231 and HcpC in *H. pylori* cells. Asterisk (^∗^) mark unspecific bands. **(B)** HcpC in *H. pylori* wt and *H. pylori hp0231::cat*. **(C)** Control of the used methodology: HP0231 in *H. pylori* wt and *H. pylori hp0231::cat*. Bacterial cultures were treated with 10% TCA, followed by alkylation with AMS. Cellular proteins including the reduced (red; DTT treated, modified with AMS) and the oxidized (ox; non-modified with AMS) controls were separated by 18% SDS-PAGE under non-reducing conditions, followed by Western-blot analysis using rabbit antibodies against HcpC or HP0231. Each lane contains proteins isolated from the same amount of bacteria. The reduced form of HcpC is not recognized by anti-HcpC serum **(B)** probably due to the large numbers of AMS particles bound to reduced form of the HcpC.

Given that HP0231 is a homodimer and the two well-characterized *E. coli* homodimeric oxidoreductases (EcDsbC and EcDsbG) function as molecular chaperones (Shao et al., 2000; Zhao et al., 2003), we examined whether HP0231 also acts as molecular chaperone by analyzing its influence on the thermal aggregation of two classical chaperone substrate proteins, CS and LUC. As EcDsbA exhibits low chaperone activity (Zheng et al., 1997), the monomeric derivative of HP0231 (HP0231m) was also included in these tests. We found that HP0231 as well as HP0231m reveal chaperone activity. HP0231 showed activity similar to dimeric EcDsbG, whereas monomeric HP0231m prevented the thermal aggregation of substrate proteins even more efficiently than both homodimeric proteins (**Figures 8D,E**). HP0231 has recently been described as a folding factor of HcpE (HP0235; Lester et al., 2015). HcpE potentially contains nine disulfide bonds, as judged from structure modeling based on the previously solved structure of HcpC (Luthy et al., 2004). It is still unclear whether HP0231 is involved in the formation of the HcpE disulfide bonds. The authors documented that the lack of HP0231 resulted in decrease of the amount of HcpE and showed the interaction between HcpE and HP0231 in *in vitro* experiments. However, they did not show that the activity of HP0231 influences the redox state of HcpE *in vivo*. To better understand the potential impact of HP0231 on the structure of Hcp proteins, we evaluated the redox state of another member of the Hcp family – HcpC. According to the data presented by Lester et al. (2015), we found that the amount of HcpC present in *H. pylori hp0231* isogenic mutant is reduced as compared to *H. pylori* wild type (**Figure 9A**). However, using the AMS trapping strategy we showed that the lack of HP0231 potentially does not influence the HcpC redox state. Notice that in the AMS trapping experiment the reduced form of HcpC is not recognized by specific antibodies, probably due to the large numbers of AMS particles bound to reduced form of the HcpC. As a control of the used methodology, we checked the redox state of HP0231 present in the same bacterial culture (**Figures 9B,C**). Additionally, the used strategy does not discriminate between correctly oxidized and misoxidized protein.

### Crystal Structure of HP0231m

**FIGURE 10:**
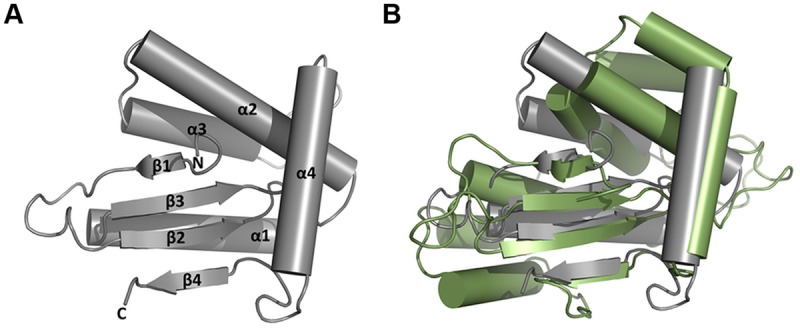
**Structural classification of ΔHP0231 (aa 132–264). (A)** Structure of ΔHP0231. **(B)** Superposition of ΔHP0231 (gray) and MtbDsbA (green; pdb:4K6X). The β1 ΔHP0231 strand forms hydrogen bonds with β3 strand, which classifies it to the DsbA_II group.

It was unexpected that HP0231m was not able to reduce insulin, even though it was active *in vivo* in *E. coli*. Its other distinguishing biochemical feature, as compared to EcDsbA, is a high chaperone activity. Thus, the HP0231m structure was solved and compared to the structures of EcDsbA and MtDsbA (**Figures 10A,B**). The HP0231m structure can be superimposed onto the full-length HP0231 structure (PDB: 3tdg) with an RMSD of 2.1 Å, and according to Dali Lite searches, it is also very similar. The β1 forms hydrogen bonds to β3, a feature observable in structures of catalytic domains of DsbG, DsbC, and also in class II DsbA-like proteins (McMahon et al., 2014). This is in agreement with our phylogenetic analysis, which showed that the DsbG/C/HP0231 branch is more closely related to class II DsbA-like proteins than to canonical class I DsbA (**Figure 1**).

## Discussion

The *H. pylori* HP0231 protein is an oxidoreductase that acts as a homodimer (Yoon et al., 2011; Roszczenko et al., 2012). So far, this unique structure for proteins involved in the oxidative pathway of disulfide bond formation has only been described for *L. pneumophila* (Kpadeh et al., 2013, 2015) and the Gram-positive *C. glutamicum* (Daniels et al., 2010). However, apart from the oxidative function, all the above proteins differ significantly in their structure, CXXC and *cis*-Pro active sites, and in their phylogenetic origin. Also, the whole set of Dsb proteins involved in the oxidative pathway varies, depending on the microorganism species. *L. pneumophila* encodes two DsbAs, (monomeric DsbA1 and dimeric DsbA2), as well as two DsbB and two DsbD proteins, whereas *H. pylori* lacks both a classical DsbB and a classical DsbD (Raczko et al., 2005; Kpadeh et al., 2015). The catalytic domain of HP0231 possesses catalytic motifs typical for canonical DsbA proteins, but evolutionarily it is most closely related to the catalytic domains of DsbG (**Figure 1**). Similarly, the highly diverged N-terminal dimerization domain is homologous to the dimerization domain of DsbG (**Figure 2**). However, in this case, the sequence similarity is very low (12% over the aligned region) and the homology relationship could only be confirmed based on sequence profile–profile comparisons (see Materials and Methods) and structural similarity (Yoon et al., 2011). The well-characterized *L. pneumophila* LpDsbA2, alike HP0231, is a dimeric protein possessing oxidoreductase activity. It must be emphasized, however, that the two proteins are unrelated. First, the catalytic domain of LpDsbA2 belongs to the type II DsbA class (McMahon et al., 2014), whereas the catalytic domain of HP0231 originates from DsbG proteins. Second, the two proteins contain different and evolutionarily unrelated dimerization domains: a coiled-coil domain in LpDsbA2 and a DsbG/C-like domain in HP0231. Considering that dimerization domains seem to occur only in the context of the β1 arrangement observed in HP0231, DsbG/C, and class II DsbA-like catalytic domains, one can speculate that it might be a prerequisite for the formation of two-domain Dsb proteins, in which a dimerization domain precedes the catalytic domain. The combination of different catalytic and dimerization domains are reflected in the protein functions. LpDsbA2 displays both an oxidizing and an isomerization activity in the native host (*L. pneumophila*), which is ensured by concerted action of the LpDsbBs and LpDsbDs proteins (Kpadeh et al., 2015). Our previous work and data presented in this paper indicated that in the native host (*H. pylori*), HP0231 is an oxidoreductase responsible for disulfide bond formation, as was proven by: evaluating its redox state, its influence on cell resistance to DTT (Roszczenko et al., 2012), cadmium sensitivity, and also confirmed by its ability to oxidize unfolded, reduced RNaseA (ruRNaseA) *in vitro*. Recently published data by Lester et al. (2015) showed that HP0231, as well as EcDsbA and the EcDsbG used as a control in this experiment, have a rather weak efficiency at refolding denatured, unfolded lysozyme under standard conditions. However, changing the reaction conditions resulted in a high oxidizing activity for HP0231. Previously lysozome has been used to study the mechanism of action of a eukaryotic protein-disulfide isomerase (PDI), capable of forming and rearranging disulfide bonds during oxidative protein folding. The specific structure of PDI (two thioredoxin domains) is responsible for its dual function (Puig and Gilbert, 1994; Puig et al., 1994). EcDsbG is not active in the test presented by Lester et al. (2015), as EcDsbG, despite its dimeric structure that is similar to EcDsbC (the main isomerase of *E. coli*), differs from DsbC by reacting preferentially with folded proteins (Heras et al., 2004), and it is mainly involved in the control of the cysteine sulfenylation level, which protects single cysteine residues from oxidation to sulfenic acid (Depuydt et al., 2009; Kadokura and Beckwith, 2010).

In *E. coli* cells, LpDsbA2 is present in the reduced form, provided by EcDsbD, and functions only as isomerase, whereas HP0231 in the same background is present in the oxidized form, acts as oxidase and does not show isomerization activity, as was proven previously and in this work by *in vitro* and *in vivo* experiments. At this point, it is worthwhile to emphasize that contrary to *L. pneumophila, H. pylori* does not encode a classical DsbD. Instead, it has shortened version of DsbD, CcdA (Cho et al., 2012). Thus, HP0231 is potentially not capable of interacting with EcDsbD.

Our recently performed *in vitro* experiments identified apocytochrome c (HP1227) as HP0231 substrate (Roszczenko et al., 2015). Oxidative folding potentially protects apocytochrome c from degradation. However, the cytochrome c maturation process required ligation of heme to reduced thiols of CXXCH motif of apocytochrome. Thus, the oxidized HP1227 is next reduced by the action of HP0377 – CcmG, an *H. pylori* Dsb protein involved in cytochrome c biogenesis (Bocian-Ostrzycka et al., 2015). We also showed that HP0377 reactivates oxidized, scrambled RNase (scRNaseA; Roszczenko et al., 2015). Since *H. pylori* does not possess a classical DsbC, HP0377 may contribute to the Dsb-related isomerization pathway *in vivo* and the close cooperation between HP0231 and HP0377 ensures the proper protein oxidative folding.

As was shown in this paper, HP0231 prevents the thermal aggregation of CS and LUC. Recently, it has been shown that HP0231 acts as folding factor for HcpE, which is consistent with its chaperone activity (Lester et al., 2015). Similarly to the data presented by Lester et al. (2015) we found that the lower amount of HcpC protein is present in the *hp0231* isogenic mutant than in *H. pylori* wild-type as a consequence of the lack of HP0231 chaperone activity. We also evaluated the influence of HP0231 on the redox state of the HcpC using AMS trapping strategy. We showed, that the lack of HP0231 did not change the HcpC redox state. However, it should be stressed that AMS trapping experiment does not distinguish between properly folding and misfolding HP0231. Thus the mechanism of the *H. pylori* Hcp oxidation still remains unclear.

The catalytic domains of both dimeric oxidoreducases (HP0231m and LpDsbA2N) are active in *E. coli* cells in an EcDsbB-dependent manner (Kpadeh et al., 2013). However, HP0231m and LpDsbA2N (a truncated form of the LpDsbA2) differ in their biochemical properties. In contrast to LpDsbA2N, HP0231m is not active in the insulin reduction assay, and additionally, it displays a high chaperone activity. These features that are untypical for a monomeric DsbA protein may reflect its origin. HP0231 originates from DsbG, the Dsb protein which is not active in the insulin reduction assay and which possesses/exhibits chaperone activity. The solved structure of the HP0231 catalytic domain (HP0231m) confirmed the phylogenetic analysis and showed that it is similar in structure to class II DsbA proteins. Members of this class, such as MtDsbA or SaDsbA (*Mycobacterium tuberculosis* or *Staphylococcus aureus* DsbA, respectively), also are not active in the insulin reduction assay (Heras et al., 2008; Chim et al., 2013). However, it should be noted that the HP0231 catalytic domain contains CXXC and *cis-*Pro motifs characteristic of class I DsbA proteins (CPHC and V*c*P). So far, the dimeric structure of oxidoreductases was considered to be crucial for their chaperone activity (Zhao et al., 2003). Thus, our data demonstrating high chaperone activity for HP0231m are rather unexpected and indicate a new, still unidentified mechanism that allows a monomeric Dsb, which originated from the dimeric DsbG, to act as a molecular chaperone.

It is still unclear how dimeric Dsb proteins involved in disulfide bond formation are reoxidized *in vivo. L. pneumophila* encodes two DsbBs, *Actinobacteria* does not encode DsbB, and *H. pylori* encodes a DsbB-like protein. We showed that activity of dimeric HP0231 is independent of EcDsbB (in *E. coli*) and in *H. pylori* the lack of DsbI slightly affects its redox state but doesn’t influence its function in motility. The mechanism of the reoxidizing process of HP0231 still needs to be clarified.

## Conclusion

Taken together, HP0231 is a unique dimeric oxidoreductase involved in disulfide bond formation. It combines oxidative functions characteristic of DsbA proteins and chaperone activity characteristic of DsbC/DsbG, and it lacks isomerization activity. Those combined untypical features result from its evolutionary origin, which is noticeable in the structure of its catalytic domain. Although the mechanism responsible for HP0231 reoxidation remains to be fully elucidated, we demonstrated that this protein is present in an oxidized form that is efficiently maintained in native host and *E. coli*. Moreover, we demonstrated that the HP0231 dimeric structure is necessary for its interaction with substrates in the native host.

## Author Contributions

EJ-K, KB-O, and AL conceived and designed the study. SD-H was responsible for phylogenetic analysis. EN and KB-O was responsible for crystallography. KB-O, AL, KD, AD, MG, and RG carried out the laboratory work. EJ-K, KB-O, and AL analyzed the data. EJ-K, KB-O, AL and J-FC wrote the manuscript. All authors read and approved the final manuscript.

## Conflict of Interest Statement

The authors declare that the research was conducted in the absence of any commercial or financial relationships that could be construed as a potential conflict of interest.
