# Supplementary material for: Functional and evolutionary analyses of Helicobacter pylori HP0231 (DsbK) protein with strong oxidative and chaperone activity characterized by a highly diverged dimerization domain
Source: Front Microbiol. 2015 Oct 8;6:1065. doi: 10.3389/fmicb.2015.01065 (PMC4597128; doi:10.3389/fmicb.2015.01065)
Supplement: Supplementary file 1 [file Table_1.PDF]

Supplementary table S1. Extended table containing strains and plasmids used in this study

Name	Relevant characteristics	Source/Ref.
<u>*Helicobacter pylori* strains</u>		
26695	*H. pylori* wild-type	ATCC
N6	*H. pylori* wild-type	(Behrens *et al.*, 2012)
PR378	N6 *hp0231::cat*	(Roszczenko *et al.*, 2012)
PR397	N6 *hp0231::cat*/pUWM397 (*hp0231*$^+$ *in trans*)	(Roszczenko *et al.*, 2012)
KBO570	*N6 hp0231::cat/pUWM570 (EcdsbA*$^+$ *in trans* under native *hp0231* promoter)	This study
KBO574	*N6 hp0231::cat/pUWM574 (hp0231m*$^+$ *in trans)*	This study
PR305	N6 *dsbI::aph*	(Roszczenko *et al.*, 2012)
KBO571	N6 *dsbI::aph*/pUWM571 (*EcdsbA in trans* under native *hp0231* promoter)	This study
KBO575	N6 *dsbI::aph*/pUWM575 *(hp0231m*$^+$ *in trans)*	This study
<u>*Escherichia coli* strains:</u>		
TG1	*supE44 hsdΔ 5 thi Δ(lac*$^-$ *proAB) F' [traD36 proAB*$^+$ *lacI*^{q} *lacZΔM15]*	(Sambrook and Russel, 2001)
BL21 (DE3)	F$^-$*ompT hsdS*$_B$*(r*$_B$$^-$*m*$_B$$^-$*) gal dcm lon*	Novagen
BL21/*EcdsbA*$^+$	BL21 carrying pET28a/*EcdsbA*	JFC Collection
BL21/*EcdsbC*$^+$	BL21 carrying pET28a/*EcdsbC*	JFC Collection
BL21/*EcdsbG*$^+$	BL21 carrying pET28a/*EcdsbG*	JFC Collection
RG2022	BL21 carrying pUWM2021 (*hcpC*$^+$)	This study
Rosetta (DE3)pLacI	F$^-$ *ompT hsdS*$_B$ *(r*$_B$*- m*$_B$*-) gal dcm* pLacIRARE (Cm^r)	Novagen
KBO2044	Rosetta carrying pUWM525 (*hp0231*$^+$)	This study
KBO2030	Rosetta carrying pUWM591(*hp0231m*$^+$)	This study
JCB816	MC1000 *phoR λ102*	(Bardwell *et al.*, 1991)
JCB817	JCB 816 *dsbA::kan1*	(Bardwell *et al.*, 1991)
JCB818	JCB 816 *dsbB::kan2*	(Bardwell *et al.*, 1991)
JCB819	JCB 816 *dsbAB::kan1,2*	(Bardwell *et al.*, 1991)
KBO519	JCB816 carrying pHel2	This study
PR501	JCB817 carrying pHel2	(Roszczenko *et al.*, 2012)
PR521	JCB818 carrying pHel2	(Roszczenko *et al.*, 2012)
KBO523	JCB819 carrying pHel2	This study
KBO520	JCB816 carrying pUWM500 (*HP0231*$^+$ *in trans)*	This study
PR503	JCB817 carrying pUWM500 (*HP0231*$^+$ *in trans)*	(Roszczenko *et al.*, 2012)
PR522	JCB818 carrying pUWM500 (*HP0231*$^+$ *in trans)*	(Roszczenko *et al.*, 2012)
KBO524	JCB819 carrying pUWM500 (*HP0231*$^+$ *in trans)*	This study
KBO576	JCB817 carrying pUWM575 (*HP0231m*$^+$ *in trans)*	This study
KBO586	JCB818 carrying pUWM575 (*HP0231m*$^+$ *in trans)*	This study
PL263	*MC1000 mdoG::kan1; dsbC::kan2*	(Leverrier *et al.*, 2011)
PL284	PL263 carrying pBAD33	(Leverrier *et al.*, 2011)
PL285	PL263 carrying JFC355 (*dsbC*$^+$ *in trans*)	(Leverrier *et al.*, 2011)
KBO2087	PL263 carrying pUWM500 (*HP0231*$^+$ *in trans)*	This study
KBO2088	PL263 carrying pUWM575 (*HP0231m*$^+$ *in trans)*	This study
<u>Plasmids:</u>		
pET28a	Km^r, IPTG inducible	Novagen
pET39b	Km^r, IPTG inducible; *EcdsbA+*	Novagen
pGEM T-Easy	Ap^r; LacZα	Promega
pHel2	Cm^r *E. coli/H. pylori* shuttle vector	(Heuermann and Haas, 1998)
pHel3	Km^r *E. coli/H. pylori* shuttle vector	(Heuermann and Haas, 1998)
pUWM389	*hp0231*$^+$ in pGEM T-Easy	(Roszczenko *et al.*, 2012)
pUWM397	*hp0231*$^+$ in pHel3	(Roszczenko *et al.*, 2012)
pUWM500	*hp0231*$^+$ in pHel2	(Roszczenko *et al.*, 2012)
pUWM568	*hp0231m*$^+$ (*hp0231* lacking dimerization domain) in pGEM T-Easy	This study
pUWM574	*hp0231m*$^+$ in pHel3	This study

pUWM575	*hp0231m*$^{+}$ in pHel2	This study
pUWM569	*EcdsbA*$^{+}$ in fusion with promoter of *hp0231* gene in pGEM T-Easy	This study
pUWM570	*EcdsbA*$^{+}$ in fusion with promoter of HP0231 gene in pHel3	This study
pUWM2020	*hcpC*$^{+}$ in pGEM T-Easy	This study
pUWM2021	*hcpC*$^{+}$ in pET39b	This study
pUWM2029	*hp0231m*$^{+}$ in pET28a	This study
pET28a/*EcdsbA*	*EcdsbA*$^{+}$ in pET28a	JFC Collection
pET28a/*EcdsbC*	*EcdsbC*$^{+}$ in pET28a	JFC Collection
pET28a/*EcdsbG*	*EcdsbG*$^{+}$ in pET28a	JFC Collection
