# Supplementary material for: Functional and evolutionary analyses of Helicobacter pylori HP0231 (DsbK) protein with strong oxidative and chaperone activity characterized by a highly diverged dimerization domain
Source: Front Microbiol. 2015 Oct 8;6:1065. doi: 10.3389/fmicb.2015.01065 (PMC4597128; doi:10.3389/fmicb.2015.01065)
Supplement: Supplementary file 2 [file Data_Sheet_1.PDF]

Supplementary material

Functional and evolutionary analyses of *Helicobacter pylori* HP0231 (DsbK) protein with strong oxidative and chaperone activity characterized by a highly diverged dimerization domain

Katarzyna M. Bocian-Ostrzycka[1], Anna M. Łasica[1$], Stanisław Dunin-Horkawicz[2], Magdalena Grzeszczuk[1], Karolina Drabik[1$], Aneta M. Dobosz[1$], Renata Godlewska[1], Elżbieta Nowak[3], Jean-Francois Collet[4], Elżbieta K. Jagusztyn-Krynicka[1#]

[1] Department of Bacterial Genetics, Institute of Microbiology, Faculty of Biology, University of Warsaw, Warsaw, Poland,
[2] Laboratory of Bioinformatics and Protein Engineering, International Institute of Molecular and Cell Biology, Warsaw, Poland,
[3] Laboratory of Protein Structure, International Institute of Molecular and Cell Biology, Warsaw, Poland,
[4] de Duve Institute, Université catholique de Louvain (UCL)/Walloon Excellence in Life Sciences and Biotechnology, Brussels, Belgium

[#] Corresponding author
Elżbieta K. Jagusztyn-Krynicka
Department of Bacterial Genetics, Institute of Microbiology, Faculty of Biology, University of Warsaw; Miecznikowa 1 str. 02-096 Warsaw, Poland
Phone: +4822 5541404; Fax: +4822 5541402
kjkryn@biol.uw.edu.pl

[$] Current addresses:
AŁ: Department of Oral Immunology and Infectious Diseases, University of Louisville School of Dentistry, Louisville, KY 40202, USA
KD: Laboratory of Bioenergetics and Biomembranes, Nencki Institute of Experimental Biology PAS, Warsaw
AD: Laboratory of Cell Signaling and Metabolic Disorders, Nencki Institute of Experimental Biology PAS, Warsaw

1. Supplementary figures and tables

1.1. Supplementary figures

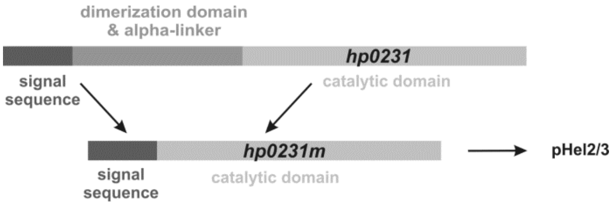

Supplementary figure S1. Schematic representation of the *hp0231* monomeric derivative – *hp0231m*. Vectors (pHel2 and pHel3) carrying *hp0231m* were constructed by a two-step PCR method. The DNA region encoding the promoter region of the *hp0231* gene with a native signal sequence (dark grey) was amplified from the chromosome of *H. pylori* 26695. Also the catalytic domain of the *hp0231* gene (light grey), including the active motif CXXC, was amplified from the chromosome of *H. pylori* 26695. Next, a mixture of the two purified products (in equal amounts) was used as a template in a single PCR reaction. Finally, the DNA region encoding *hp0231m* was transferred into pHel2 and pHel3.

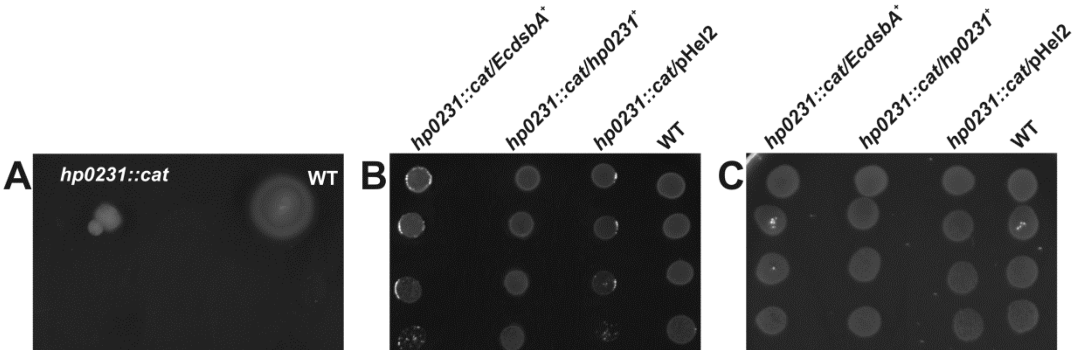

Supplementary figure S2. EcDsbA is not active in *H. pylori hp0231::cat* mutant cells. As a positive control, *H. pylori* N6 *hp0231::cat* was transformed with pHel2 carrying the native *hp0231* gene. **Panel A**: motility assay. Bacterial motility was monitored after 4 days of incubation on 0.35% MH-agar plates containing 10% FCS. The *hp0231::cat* and *hp0231::cat/EcdsbA*$^+$ strains are non-motile. **Panels B and C:** cadmium sensitivity assay. Exponentially growing *H. pylori* wt, *hp0231::cat* mutant, *hp0231::cat/hp0231*$^+$ and *hp0231::cat/EcdsbA*$^+$ strain cultures were ten-fold serially diluted and spotted on BA plates

without (**panel B**) or with (**panel C**) 8 μM $CdCl_2$, and incubated at 37°C. The mutant shows reduced growth after 3 days of incubation on plates containing cadmium chloride.

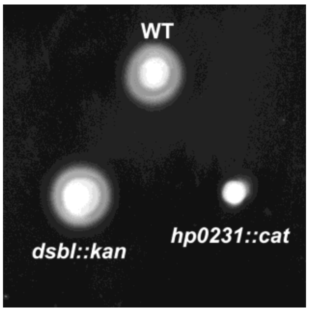

Supplementary figure S3. *HpdsbI::kan* mutation does not influence *H. pylori* motility. The figure presents motility of *H. pylori* N6 strains: wt, *hp0231::cat* and *HpdsbI::kan.* Bacterial motility was monitored after 4 days of incubation on 0.35% MH-agar plates containing 10% FCS. Only the *hp0231* mutant strain is non-motile.

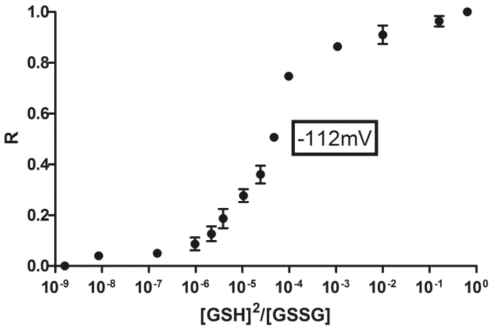

Supplementary figure 4. Redox equilibrium of *H. pylori* HP0231m with glutathione is the same as HP0231. The fraction of reduced (R) HP0231m was determined using the specific HP0231 fluorescence at 330 nm. The graph represent the average of three independent experiments, the bars represent standard deviation.

1.2. Supplementary tables

Supplementary table S1. Extended table containing strains and plasmids used in this study

Name	Relevant characteristics	Source/Ref.
Helicobacter pylori strains		
26695	*H. pylori* wild-type	ATCC
N6	*H. pylori* wild-type	(Behrens *et al.*, 2012)
PR378	N6 *hp0231::cat*	(Roszczenko *et al.*, 2012)
PR397	N6 *hp0231::cat*/pUWM397 (*hp0231*$^+$ *in trans*)	(Roszczenko *et al.*, 2012)
KBO570	*N6 hp0231::cat/pUWM570 (EcdsbA*$^+$ *in trans* under native *hp0231* promoter)	This study
KBO574	*N6 hp0231::cat/pUWM574 (hp0231m*$^+$ *in trans)*	This study
PR305	N6 *dsbI::aph*	(Roszczenko *et al.*, 2012)
KBO571	N6 *dsbI::aph*/pUWM571 (*EcdsbA in trans* under native	This study

	hp0231 promoter)	
KBO575	N6 *dsbI::aph*/pUWM575 *($hp0231m^+$ in trans)*	This study
<u>*Escherichia coli* strains:</u>		
TG1	*supE44 hsdΔ 5 thi Δ(lac⁻ proAB) F′ [traD36 proAB⁺ lacI^q lacZΔM15]*	(Sambrook and Russel, 2001)
BL21 (DE3)	F⁻*ompT hsdS_B(r_B⁻m_B⁻) gal dcm lon*	Novagen
BL21/*EcdsbA⁺*	BL21 carrying pET28a/*EcdsbA*	JFC Collection
BL21/*EcdsbC⁺*	BL21 carrying pET28a/*EcdsbC*	JFC Collection
BL21/*EcdsbG⁺*	BL21 carrying pET28a/*EcdsbG*	JFC Collection
RG2022	BL21 carrying pUWM2021 (*$hcpC^+$*)	This study
Rosetta (DE3)pLacI	F⁻ *ompT hsdS_B (r_B- m_B-) gal dcm* pLacIRARE (Cm^r)	Novagen
KBO2044	Rosetta carrying pUWM525 (*$hp0231^+$*)	This study
KBO2030	Rosetta carrying pUWM591(*$hp0231m^+$*)	This study
JCB816	MC1000 *phoR λ102*	(Bardwell *et al.*, 1991)
JCB817	JCB 816 *dsbA::kan1*	(Bardwell *et al.*, 1991)
JCB818	JCB 816 *dsbB::kan2*	(Bardwell *et al.*, 1991)
JCB819	JCB 816 *dsbAB::kan1,2*	(Bardwell *et al.*, 1991)
KBO519	JCB816 carrying pHel2	This study
PR501	JCB817 carrying pHel2	(Roszczenko *et al.*, 2012)
PR521	JCB818 carrying pHel2	(Roszczenko *et al.*, 2012)
KBO523	JCB819 carrying pHel2	This study
KBO520	JCB816 carrying pUWM500 (*$HP0231^+$ in trans)*	This study
PR503	JCB817 carrying pUWM500 (*$HP0231^+$ in trans)*	(Roszczenko *et al.*, 2012)
PR522	JCB818 carrying pUWM500 (*$HP0231^+$ in trans)*	(Roszczenko *et al.*, 2012)
KBO524	JCB819 carrying pUWM500 (*$HP0231^+$ in trans)*	This study
KBO576	JCB817 carrying pUWM575 (*$HP0231m^+$ in trans)*	This study
KBO586	JCB818 carrying pUWM575 (*$HP0231m^+$ in trans)*	This study
PL263	*MC1000 mdoG::kan1; dsbC::kan2*	(Leverrier *et al.*, 2011)
PL284	PL263 carrying pBAD33	(Leverrier *et al.*, 2011)
PL285	PL263 carrying JFC355 (*$dsbC^+$ in trans*)	(Leverrier *et al.*, 2011)
KBO2087	PL263 carrying pUWM500 (*$HP0231^+$ in trans)*	This study
KBO2088	PL263 carrying pUWM575 (*$HP0231m^+$ in trans)*	This study
<u>Plasmids:</u>		
pET28a	Km^r, IPTG inducible	Novagen
pET39b	Km^r, IPTG inducible; *EcdsbA+*	Novagen
pGEM T-Easy	Ap^r; LacZα	Promega
pHel2	Cm^r *E. coli/H. pylori* shuttle vector	(Heuermann and Haas, 1998)
pHel3	Km^r *E. coli/H. pylori* shuttle vector	(Heuermann and Haas, 1998)
pUWM389	*$hp0231^+$* in pGEM T-Easy	(Roszczenko *et al.*, 2012)
pUWM397	*$hp0231^+$* in pHel3	(Roszczenko *et al.*, 2012)
pUWM500	*$hp0231^+$* in pHel2	(Roszczenko *et al.*, 2012)
pUWM568	*$hp0231m^+$* (*hp0231* lacking dimerization domain) in pGEM T-Easy	This study
pUWM574	*$hp0231m^+$* in pHel3	This study
pUWM575	*$hp0231m^+$* in pHel2	This study
pUWM569	*$EcdsbA^+$* in fusion with promoter of *hp0231* gene in pGEM T-Easy	This study
pUWM570	*$EcdsbA^+$* in fusion with promoter of HP0231 gene in pHel3	This study
pUWM2020	*$hcpC^+$* in pGEM T-Easy	This study
pUWM2021	*$hcpC^+$* in pET39b	This study
pUWM2029	*$hp0231m^+$* in pET28a	This study
pET28a/*EcdsbA*	*$EcdsbA^+$* in pET28a	JFC Collection
pET28a/*EcdsbC*	*$EcdsbC^+$* in pET28a	JFC Collection
pET28a/*EcdsbG*	*$EcdsbG^+$* in pET28a	JFC Collection
