# Supplementary figures and images for: Functional and evolutionary analyses of Helicobacter pylori HP0231 (DsbK) protein with strong oxidative and chaperone activity characterized by a highly diverged dimerization domain
Source: Front Microbiol. 2015 Oct 8;6:1065. doi: 10.3389/fmicb.2015.01065 (PMC4597128; doi:10.3389/fmicb.2015.01065)

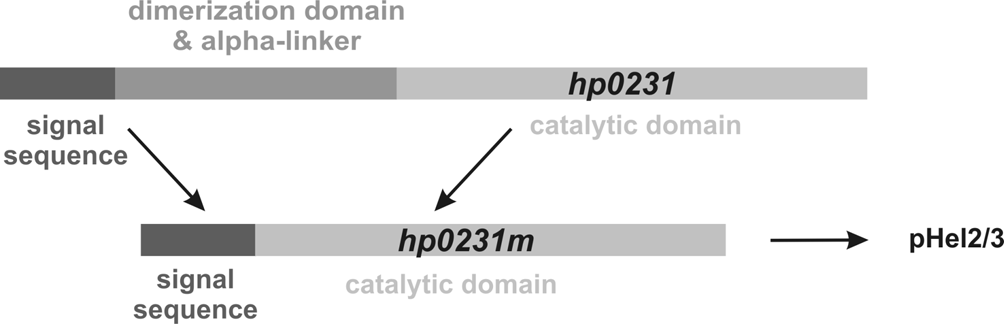

Supplement: Supplementary file 3 [file Image_1.TIF]

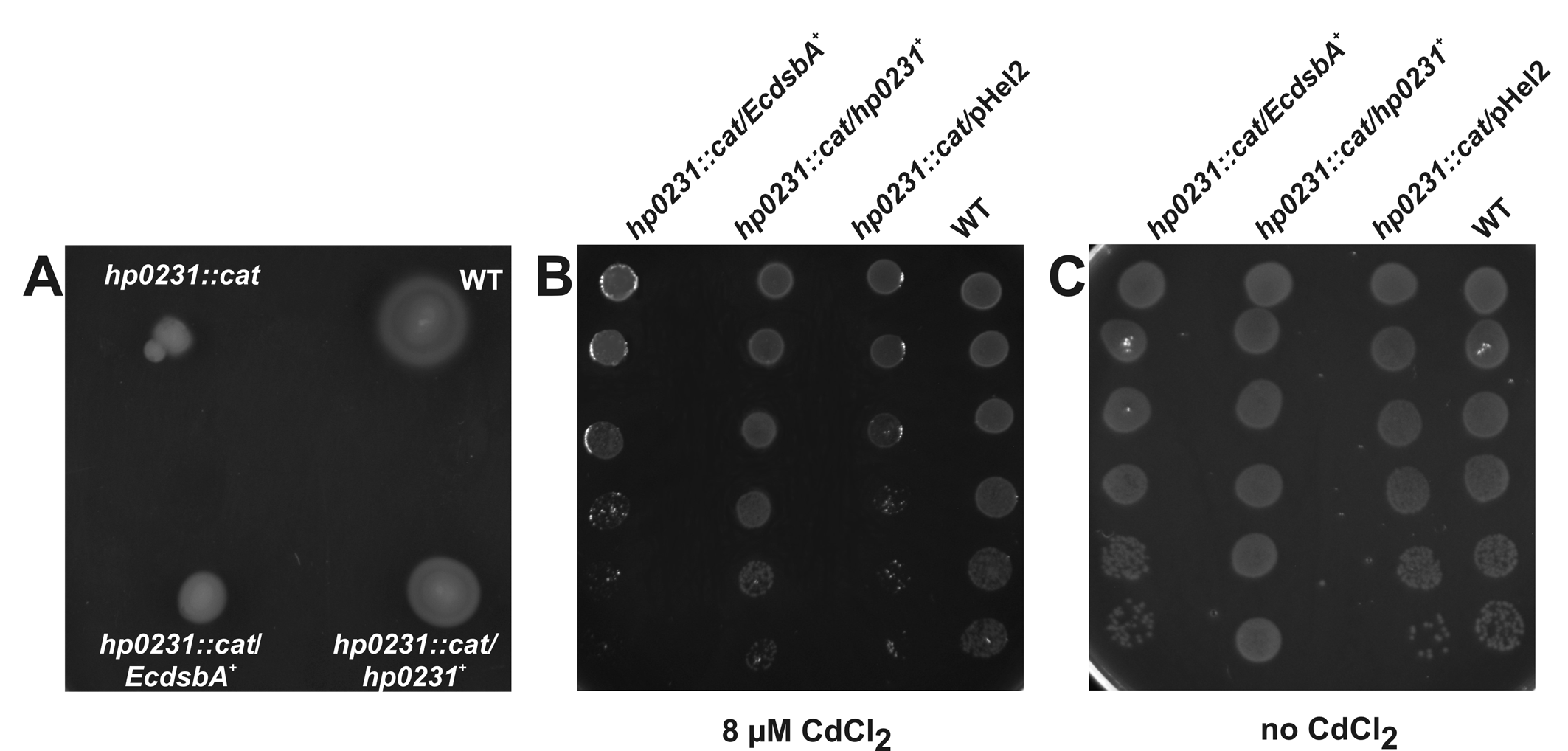

Supplement: Supplementary file 4 [file Image_2.TIF]

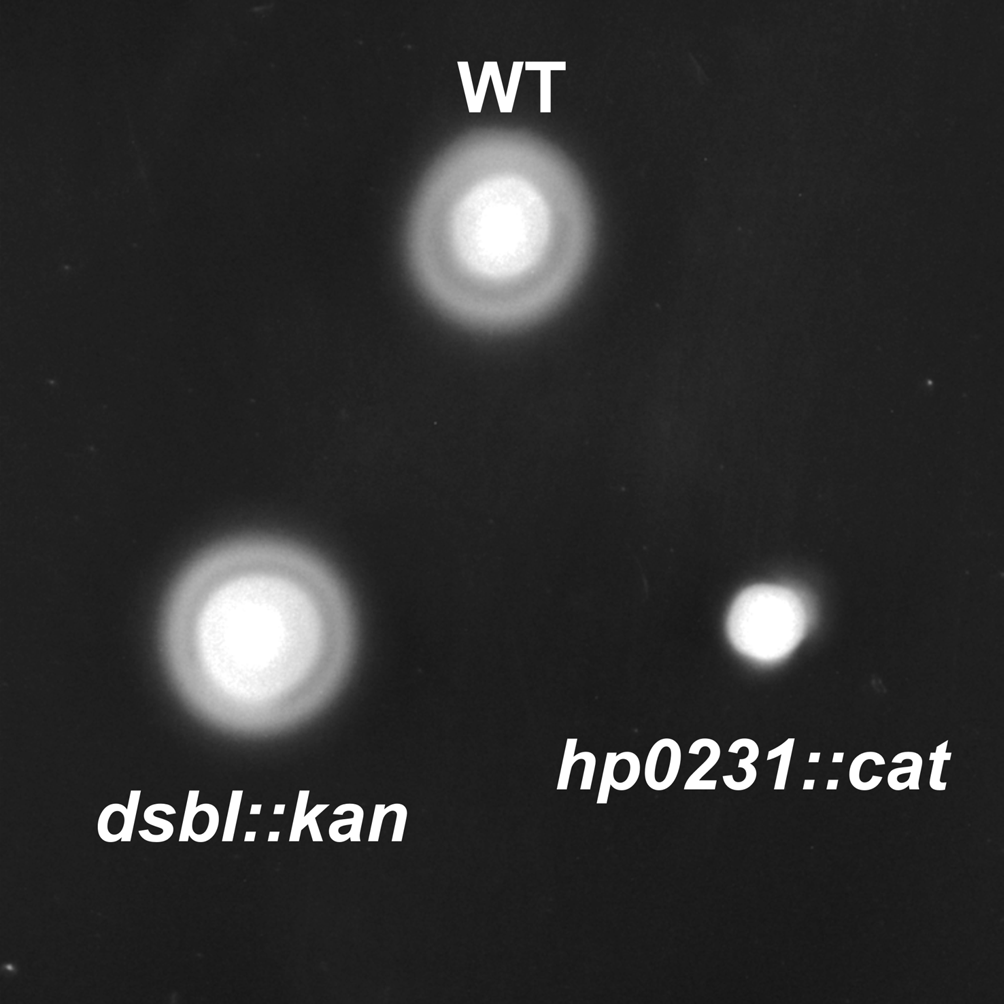

Supplement: Supplementary file 5 [file Image_3.TIF]

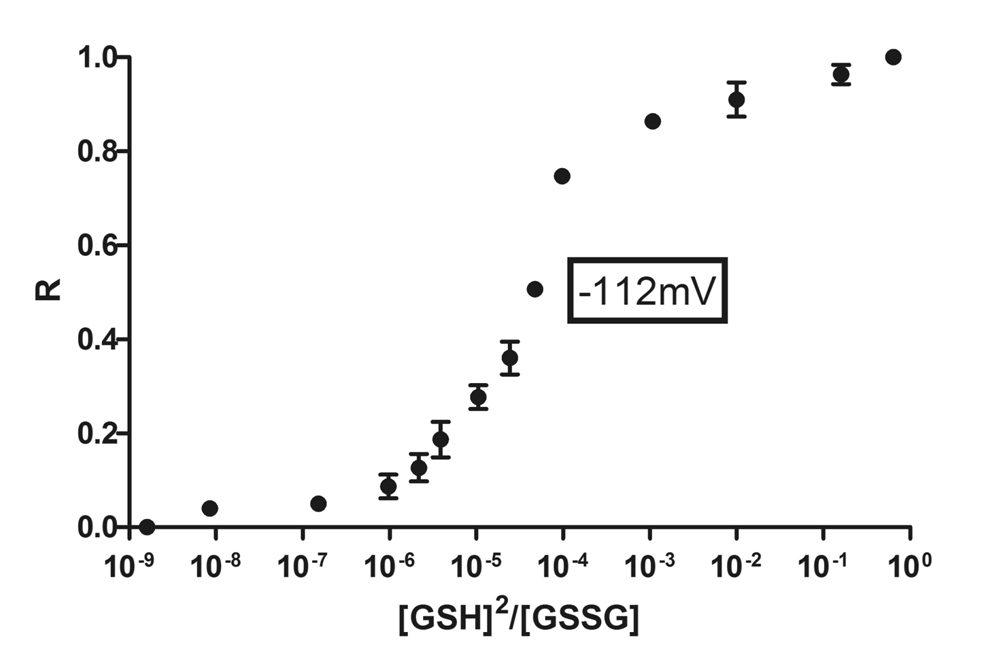

Supplement: Supplementary file 6 [file Image_4.TIF]
